# Environmental conditions play a key role in controlling the composition and diversity of Colombian biocrust microbiomes

**DOI:** 10.3389/fmicb.2024.1236554

**Published:** 2024-04-25

**Authors:** Ana Giraldo-Silva, Caroline A. Masiello

**Affiliations:** ^1^Department of Science, Ecology Group and Institute for Multidisciplinary Research in Applied Biology, Public University of Navarre (UPNA), Pamplona, Spain; ^2^Department of Earth, Environmental and Planetary Sciences, Rice University, Houston, TX, United States

**Keywords:** biological soil crust, biocrust diversity, biocrust composition, cyanobacteria, soil microbiomes, environmental conditions, Colombia-South America

## Abstract

Drylands soils worldwide are naturally colonized by microbial communities known as biocrusts. These soil microbiomes render important ecosystem services associated with soil fertility, water holding capacity, and stability to the areas they cover. Because of the importance of biocrusts in the global cycling of nutrients, there is a growing interest in describing the many microbial configurations these communities display worldwide. However, comprehensive 16S rRNA genes surveys of biocrust communities do not exist for much of the planet: for example, in the continents of South America and the northern part of Africa. The absence of a global understanding of biocrust biodiversity has lead us to assign a general importance to community members that may, in fact, be regional. Here we report for the first time the presence of biocrusts in Colombia (South America) through 16S rRNA genes surveys across an arid, a semi-arid and a dry subtropical region within the country. Our results constitute the first glance of the Bacterial/Archaeal communities associated with South American biocrust microbiomes. Communities where cyanobacteria other than *Microcoleus vaginatus* prevail, despite the latter being considered a key species elsewhere, illustrate differentiable results in these surveys. We also find that the coastal biocrust communities in Colombia include halo-tolerant and halophilic species, and that niche preference of some nitrogen fixing organisms deviate from previously described global trends. In addition, we identified a high proportion (ranging from 5 to 70%, in average) of cyanobacterial sequences that did not match any formally described cyanobacterial species. Our investigation of Colombian biocrusts points to highly diverse communities with climatic regions controlling taxonomic configurations. They also highlight an extensive local diversity to be discovered which is central to better design management and restoration strategies for drylands soils currently undergoing disturbances due to land use and global warming. Finally, this field study highlights the need for an improved mechanistic understanding of the response of key biocrust community members to changes in moisture and temperature.

## 1 Introduction

Plant interspaces in arid and semiarid areas (hereafter drylands) are typically colonized by microbial assemblages known as biological soil crusts (biocrusts; see Garcia-Pichel, 2003, for a primer, and Belnap et al., 2016, for a monograph). These communities form when different proportions of cyanobacteria (Yeager et al., 2007; Garcia-Pichel and Wojciechowski, 2009), algae (Samolov et al., 2020), archaea (Soule et al., 2009; Marusenko et al., 2013), other bacteria (Nagy et al., 2005), fungi (Bates et al., 2012), and sometimes mosses and lichens (Ullmann and Büdel, 2001) associate with soil particles creating an erosion resistant layer in the top few millimeters of the soil. Our knowledge of the biodiversity, distribution, ecology, and physiology of biocrust communities has grown in the last decades driven by the need to understand the central ecosystem services they bring locally and globally. At the local scale, biocrust presence provides soil resistance against wind (Zhang et al., 2006) and water erosion (Gaskin and Gardner, 2001), increases soil nutrient availability through atmospheric carbon (Sancho et al., 2016) and nitrogen fixation (Barger et al., 2016), and regulates soil hydrology (Chamizo et al., 2016). At the larger scale, these microbial assemblages are key actors in the global cycling of C and N. They cover approximately 12% of the land worldwide (Rodríguez-Caballero et al., 2018), where they are responsible for about 15% of the net primary production of terrestrial vegetation and nearly 50% of the nitrogen that is annually fixed on the Earth’s continental surface (Elbert et al., 2012).

Drylands will most likely increase over the next decades due to ongoing climate change (Seager and Vecchi, 2010) and current and future desertification (Nkonya et al., 2015), potentially increasing the area of influence of biocrust microbiomes. Therefore, understanding their current geographic extension and microbial configuration, especially in understudied areas, is fundamental to widening our understanding of their diversity and functions across terrestrial ecosystems. Describing the microbial diversity from biocrust communities through next generation sequencing has been the focus of multiple studies over the last 10 years. However, the vast majority of this research targeted communities from North America (Garcia-Pichel et al., 2013; Couradeau et al., 2016; Fernandes et al., 2018; Giraldo-Silva et al., 2019), Europe (Williams et al., 2016; Muñoz-Martín et al., 2018), Australia (Moreira-Grez et al., 2019), and Asia (Zhang et al., 2016), leaving multiple, very large regions such as South America and most of Africa yet to be explored. The presence of biocrust communities in the South American continent have been reported in Peru (Rengifo and Arana, 2017), Chile (Wang et al., 2017; Baumann et al., 2018; Samolov et al., 2020), Bolivia (Knudsen and Flakus, 2016), Argentina (Tabeni et al., 2014; Velasco-Ayuso et al., 2019), and Brazil (Machado-de-Lima et al., 2019; Machado de Lima et al., 2021), but the majority of these studies were limited to morphological and visual descriptions of the communities, and focused only in the phototrophic members of the community. Although two studies from Brazil used next generation sequencing to explore biocrusts across different biomes, their results were constrained only to the cyanobacterial components of the community. A comprehensive description of the biocrust prokaryotic community is not yet available for any site in South America.

Here we sequenced the 16S rRNA genes from field communities to describe the microbial diversity present in biocrust microbiomes from three climatic regions in Colombia (South America): arid, semi-arid and dry sub-humid. We also tested relationships between the observed variability in biocrust community composition and multiple climatic variables of temperature and precipitation in an effort to better understand driving forces of biocrust microbial configurations across the studied sites. Finally, we performed a meta-analysis comparing the cyanobacterial community in biocrusts communities across North and South America. Our investigation represents the first report on Bacterial/Archaeal communities (including cyanobacteria) of these soil microbiomes in one of the most understudied regions worldwide.

## 2 Materials and methods

### 2.1 Sampling

**Figure 1:**
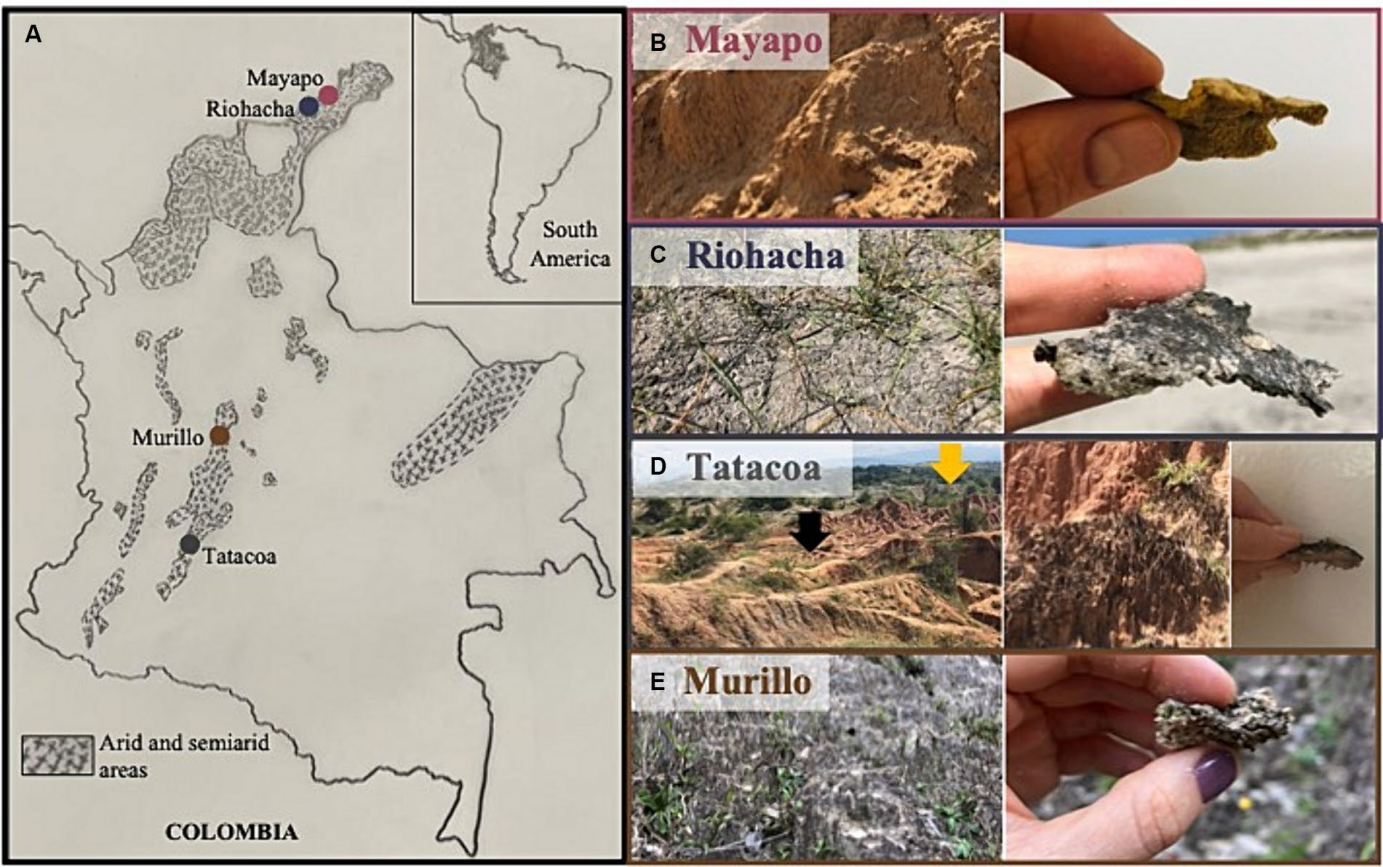
Sampling sites for Colombian biocrusts. **(A)** Left panel shows a map of Colombia localizing the major sampling regions. Shaded areas represent arid and semi-arid regions within the country. Right panels show a close view and a zoom in of biocrusted soils from **(B)** Mayapo and **(C)** Riohacha in the Guajira desert, **(D)** Los Hoyos (gray area in the back of the picture – yellow arrow), and Cusco (orange area in the front of the picture – black arrow) in the Tatacoa desert, and **(E)** Murillo in Tolima.

We sampled biocrusts at nine locations within Colombia in three climatic regions (Figure 1A): arid, semi-arid and dry sub-humid. Four of the collection sites are in the arid region of the Guajira desert: Mayapo 1 and 2 (Mayapo; Figure 1B), and Riohacha 1 and 2 (Riohacha; Figure 1C). Four other sites are in the semi-arid area of the Tatacoa desert: Tatacoa Cusco 1 and 2, and Tatacoa Los Hoyos 1 and 2 (Tatacoa; Figure 1D). One last site is located in the dry sub-humid region of Murillo in Tolima (Murillo; Figure 1E). At an elevation between 0 and 450 m.a.s.l., the Guajira desert is the largest arid area in the country. It is located in the northernmost part of South America, on the Caribbean sea and bordering Venezuela, covering most of the La Guajira peninsula in Colombia (Figure 1A – Mayapo and Riohacha). This geographic area hosts the Wayúu people, a matriarchal tribe that constitutes one of the biggest and most representative native Colombian populations (Molano Campuzano, 1964). The climate in the area is dry. High daily temperatures, which oscillate between 27 and 40°C, are cooled by the northeast trade winds that blow most of the year. Rainfall is scarce and generally occurs in the months of September, October and November (IGAC, 2011). The Tatacoa desert, located at 900 m.a.s.l., represents the second largest semi-arid area in Colombia. It extends over 335 km^2^ in a dry tropical region in central Colombia where red and grey colored rocky labyrinths (Figure 1D – red represented by black arrow – Cusco area) (Figure 1D – grey represented by yellow arrow – Los Hoyos area) are observed along the area’s landscape (Figure 1D). Temperature oscillates between 28 and 42°C during the day and between 16 and 24°C at night, and the area receives annually ~1,100 mm of precipitation (Universidad Surcolombiana, D. General de Investigaciones, 2001). Murillo is a dry sub-humid area located in the Tolima region at an elevation of 2,950 m.a.s.l. During the course of the year, the temperature generally varies from 6°C to 17°C and rarely drops below 3°C or rises above 19°C. The area has two rainy periods (March to May and October to November) and two dry seasons (January to February and December) (Cortolima, 1998). Site coordinates and their respective mean annual temperature and precipitation are found in Table 1.

**Table 1:** Biological soil crust sampling localities in Colombia.

**Desert/Region**	**Site ID**	**Coordinates in decimal**	**Mean annual temperature in °C**	**Mean annual precipitation in mm**	**Elevation in m**
La Guajira	Riohacha	11.551642, −72.912818	28.2	578	1.7
	Mayapo	11.646914, −72.786156	28.4	501	1.3
La Tatacoa	Los Hoyos	3.227972, −75.139442	27.5	1,216	489.7
	Cusco	3.230800, −75.169300	27.1	1,275	433.9
Tolima	Murillo	4.850400, −75.177856	11.4	2060	2898.6

Mean annual temperature and mean annual precipitation values were extracted from WorldClim – Global Climate Data – version 2 (http://www.worldclim.org; Fick and Hijmans, 2017).

We collected dry biocrusted soils in triplicates using 60 mm diameter x 15 mm high Petri dishes between December 2021 and January 2022. Petri dishes were placed upside down on the ground and pressed gently into biocrusted soils until the targeted microbial community touched the bottom of the Petri dish and its total height was filled with subcrusted soil (soil underneath biocrust), ensuring microbial communities were kept intact. Then Petri dishes were sealed using laboratory tape, stored in dry, dark, and low humidity conditions (11–15 RH%) and were transported to the University of Tolima in Ibagué (Colombia) for further processing.

### 2.2 Microbial community structure: DNA extraction, 16S rRNA genes sequencing, bioinformatics, and statistical analysis

Using a 1 cm diameter, 0.5 cm deep metal corer, we subsampled, in triplicates and for each of the nine locations the biocrusted part of the sampled soils (Figure 1, crust layer where particles are glued to each other). We then extracted DNA from each replicate using the Powersoil Pro extraction kit (QIAGEN, Hilden, Germany) according to manufacturer’s instructions. The V4 region of 16S rRNA genes was amplified using general bacterial primers 515F/806R to determine the biocrust’s microbial community structure through next-generation sequencing according to the protocols within Caporaso et al. (2011). The resulted microbial library was loaded in a MiSeq Illumina sequencer, adding custom 16S rRNA genes sequencing primers (Caporaso et al., 2011) on a paired-ends sequencing flow cell 2 (2 × 250 bp).

We used Qiime2 to perform different microbiome analysis on the raw DNA sequencing data obtained from Illumina. Obtained paired-end reads were demultiplexed, and a feature table containing representative sequences or ASV’s (amplicon sequence variants) and their frequency of occurrence was attained using the DADA2 plugin (Callahan et al., 2016) available in Qiime2 version 2022.2 (Caporaso et al., 2010). Preliminary taxonomic assignments were done with the RDP (Ribosomal Database Project) classifier (Wang et al., 2007), and representative sequences were then aligned against the Greengenes v13_8_9 database core reference alignment (McDonald et al., 2012). At this point, all ASV’s classified as chloroplasts were filtered out from the complete database. ASV’s were then grouped by phyla and their relative proportions were calculated to determine the bacterial community structure of each of the sampled biocrust communities. Subsequently, cyanobacterial, and non-cyanobacterial ASV’s and their counts were split into separate feature tables. Cyanobacterial ASV’s were further curated via Cydrasil3 (Roush et al., 2021),^1^ and the resulting table was used to describe the cyanobacterial community structure of biocrusts in Colombia. For diversity analysis, data was rarefied to a sampling depth of 3,150 to account for unequal sequencing depth using the alpha-rarefaction function from Qiime2. Rarefaction curves (Supplementary Figure S1) showed that to this sampling depth all samples reached a plateau, suggesting that adequate 16S rRNA genes diversity was accessed with our sequencing effort and allowing us to keep all samples and replicates sequenced for this study (Supplementary Figure S1). To describe the structure within each studied biocrust community, the number of observed ASV’s (richness), the Shannon index (quantitative measure of community richness and evenness), and the Pielou’s evenness index (measure of how species are evenly distributed in a community), as well as statistical differences (Kurskal Wallis), were calculated using the diversity metrics plugin available in Qiime2 version 2022.2. Then, biocrust bacterial community compositions were compared using principal coordinate analysis (PCoA) in R, based on Bray-Curtis pairwise distances calculated from the Hellinger transformed data (R Development Core Team, 2020). Significance between groups was tested on the Bray-Curtis matrix using the Permanova test included in the R vegan package (Oksanen et al., 2018). PCoA was constructed at the ASV’s level.

### 2.3 Microbial community composition of biocrusts across the Americas

In an attempt to compare the microbial composition of biocrust communities across the Americas (North and South America), we conducted a meta-analysis of all bacterial 16S rRNAgenes tallies available publicly. We performed a literature search, in English and in Spanish including the name of each of the countries in the Americas and the words: biocrust, biological soil crust, DNA sequencing, and 16S rRNA sequencing for the English search; and biocostras, costras biológicas, secuenciación de ADN, and 16S ARNr for the search in Spanish. We then used the accession number and the SRA toolkit from NCBI (The NCBI C++ Toolkit)^2^ to retrieve the raw sequenced data from the environmental biocrust surveys found in our literature search. A total of 121 locations distributed across four countries (USA: 28 locations, Mexico: 2, Colombia: 28 and Brazil: 63), were included in the meta-analysis. A complete list of biocrust surveys can be found in Supplementary Table S1.

A feature table for each dataset was obtained using the DADA2 plugin (Callahan et al., 2016) available in Qiime2 version 2022.2 (Caporaso et al., 2010). Before taxonomy was assigned, we used the merged table option from Qiime2 to combine the obtained feature tables and representative set of sequences. Tables were merged based on the primers set used for Illumina sequencing in each of the studies included in the meta-analysis. Accordingly, feature tables from USA (Couradeau et al., 2016; Fernandes et al., 2018; Giraldo-Silva et al., 2019; Nelson et al., 2022), Mexico (Becerra-Absalón et al., 2019) and Colombia (this study) were merged into a single table (Primer set: general bacterial 515F; 806R) and the two studies from Brazil (Machado-de-Lima et al., 2019; Machado de Lima et al., 2021) were merged in a separate table (Primer set: cyanobacterial specific CYA359 and 781a/d). We then curated the cyanobacterial taxonomy of each of the newly merged tables (merged by countries based on primer sets) by means of phylogenetic placements using Cydrasil 3 (Roush et al., 2021). The final table containing all locations and the frequency of occurrence of the different cyanobacterial taxonomical units was then manually merged in excel 16.64 at the genus or species level (when possible). Because environmental biocrusts from Brazil were sequenced with cyanobacterial-specific primers instead of general bacterial primers, our meta-analysis results only include cyanobacterial communities, thus, no comparisons at the bacterial level were performed between biocrust communities across the Americas.

We then compared the cyanobacterial community compositions from 121 biocrust communities in the American continent using principal coordinate analysis (PCoA) in R, based on Bray-Curtis pairwise distances calculated from the Hellinger transformed data (R Development Core Team, 2020). Significance between groups was tested on the Bray-Curtis matrix using the Permanova test included in the R vegan package (Oksanen et al., 2018). We also run a SIMPER analysis (PRIMER v7 – Clarke and Gorley, 2015) to determine the average dissimilarity percentage along with its cyanobacterial drivers in cyanobacterial biocrust communities among the different countries. PCoA and SIMPER analysese were performed at the ASV’s level.

### 2.4 Environmental drivers of biocrust microbiomes diversity

In an effort to relate biocrust microbiomes with environmental variables as a drivers of the spatial patterns observed in field microbial communities, we performed redundancy analysis (RDA). We considered 10 environmental variables related to temperature and precipitation: mean annual temperature, mean annual temperature during the wettest quarter of the year, mean annual temperature during the driest quarter of the year, mean annual temperature during the warmest quarter of the year, mean annual temperature during the coldest quarter of the year, mean annual precipitation, mean annual precipitation during the wettest quarter of the year, mean annual precipitation during the driest quarter of the year, mean annual precipitation during the warmest quarter of the year, and mean annual precipitation during the coldest quarter of the year. We downloaded data from WorldClim - Global Climate Data – version 2^3^ (Fick and Hijmans, 2017) and extracted it in R using the raster package (Hijmans, 2015). Environmental variables were calculated from downloaded monthly climate data for minimum, mean, and maximum temperature and for precipitation for 1970–2000. RDA analyses were assessed separately for only Colombian biocrusts and also for all biocrusts communities included in the meta-analysis (USA, Mexico, Colombia, and Brazil). In both cases, a Bray-Curtis matrix obtained from the Hellinger transformed sequenced data (see bioinformatic analysis) was used as input for the biological dataset while a Euclidean distance matrix on the normalized environmental variables was used as the input for the environmental dataset. Previous to summarizing the observed spatial biocrust microbiomes patterns throughout RDA plots, a subsequential test was run to assess which environmental variables contributed significantly to such configuration. Analyses were performed in PRIMER v7 (Clarke and Gorley, 2015) and Origin(Pro) v 2022 (OriginLab Corporation, Northampton, MA, USA). A complete list of the environmental variables included in our analysis can be found in Supplementary Table S1.

## 3 Results

### 3.1 Microbial diversity within Colombian biocrusts

**Figure 2:**
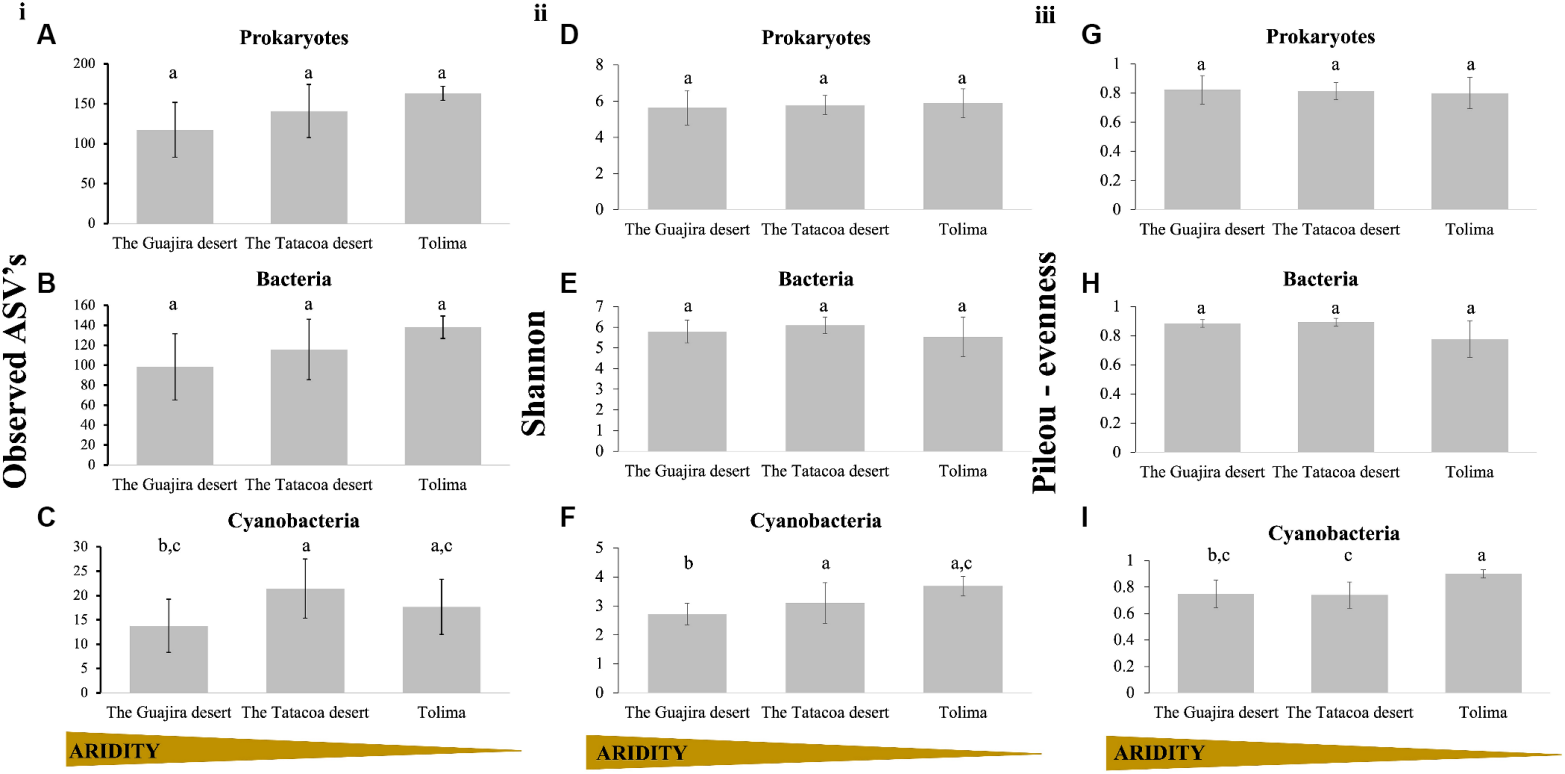
Alpha diversity of biocrust communities within Colombia. **(i)** Corresponds to Richness, panel **(ii)** corresponds to Shannon’s diversity calculations and panel **(iii)** corresponds to Pileou’s evenness. Results are displayed by evolutionary group. **(A,D,G)** Prokaryotes, **(B,E,H)** Bacteria (excluding cyanobacteria), and **(C,F,I)** Cyanobacteria only. Biocrust communities were grouped by climatic region (Guajira *n* = 12, Tatacoa *n* = 12, and Tolima *n* = 3), and are plotted, left to right, from the driest to the wettest locations. Small letters represent significance between sites (Kruskal Wallis pairwise test).

Microbial diversity within biocrusts communities (alpha-diversity) was examined through the number of observed ASV’s, the Shannon diversity index and the Pielou’s evensess index. Results are displayed by evolutionary groups (Prokaryotes as a unique group, and prokaryotes split into bacterial, and cyanobacterial communities) and by climatic region (arid: The Guajira desert, semi-arid: The Tatacoa desert and dry sub-humid: Tolima; Table 1). Richness of the prokaryotic community taken as a whole slightly increased with increasing precipitation (Observed ASV’s; Kruskal-Wallis *H* = 5.84, *p =* 0.054; Figure 2Ai). A similar trend was observed when only bacterial sequences (without cyanobacterial reads), were analyzed; however, these differences were not statistically supported (Observed ASV’s; Kruskal–Wallis H = 4.44, *p =* 0.108; Figure 2Bi). When only cyanobacterial communities were considered, communities from the driest region (The Guajira desert) were overall less rich than those from semi-arid and dry sub-humid regions, but richness did not continue increasing with precipitation (Figure 2Ci). Instead, communities from the semi-arid region of The Tatacoa desert were significantly richer than those from The Guajira desert (arid region; Kruskal Wallis pairwise test; *H* = 8.65, *p =* 0.003), and slightly richer than those from Tolima (dry sub-humid region), yet these differences were not statistically supported (Kruskal Wallis pairwise test; *H* = 0.75; *p =* 0.384). When Shannon diversity index was calculated, both prokaryotic communities as a whole and bacterial-only communities were equally diverse across climatic regions (Prokaryotes: Kruskal-Wallis H: 0.01, *p =* 0.99, Figure 2Dii; Bacteria only: Kruskal-Wallis *H*: 2.46, *p =* 0.291, Figure 2Eii). Diversity within cyanobacterial communities significantly increased with precipitation regime (Kruskal–Wallis *H*: 8.01, *p =* 0.018, Figure 2Fii). Cyaobacterial communities from Tatacoa and Tolima (semi-arid and dry sub-humid regions) were significantly more diverse than those from the Guajira desert (driest region; Kruskal Wallis pairwise test; H = 4.05 and 6.88; *p =* 0.04 and 0.008, respectively, Figure 2Dii), and equally diverse among them (Kruskal Wallis pairwise test; *H* = 0.75; *p =* 3.86, Figure 2Eii). In addition, Pileou’s evenness of both prokaryotes and bacterial communities were equal independently of location (Kruskal Wallis pairwise test; *p =* 0.70, Figures 2G,Hiii). However, cyanobacterial communities from the wettest location (Tolima) were significantly more even than those from the Tatacoa and the Guajira desert (Kruskal Wallis pairwise test; *p =* 0.014 and 0,0013, respectively, Figure 2Iiii). In sum, cyanobacterial communities from driest locations were significantly less rich and less even compared to those from less dry locations, suggesting that their diversity in Colombian biocrusts seems to increase with increasing water availability.

### 3.2 Microbial structure of Colombian biocrusts

**Figure 3:**
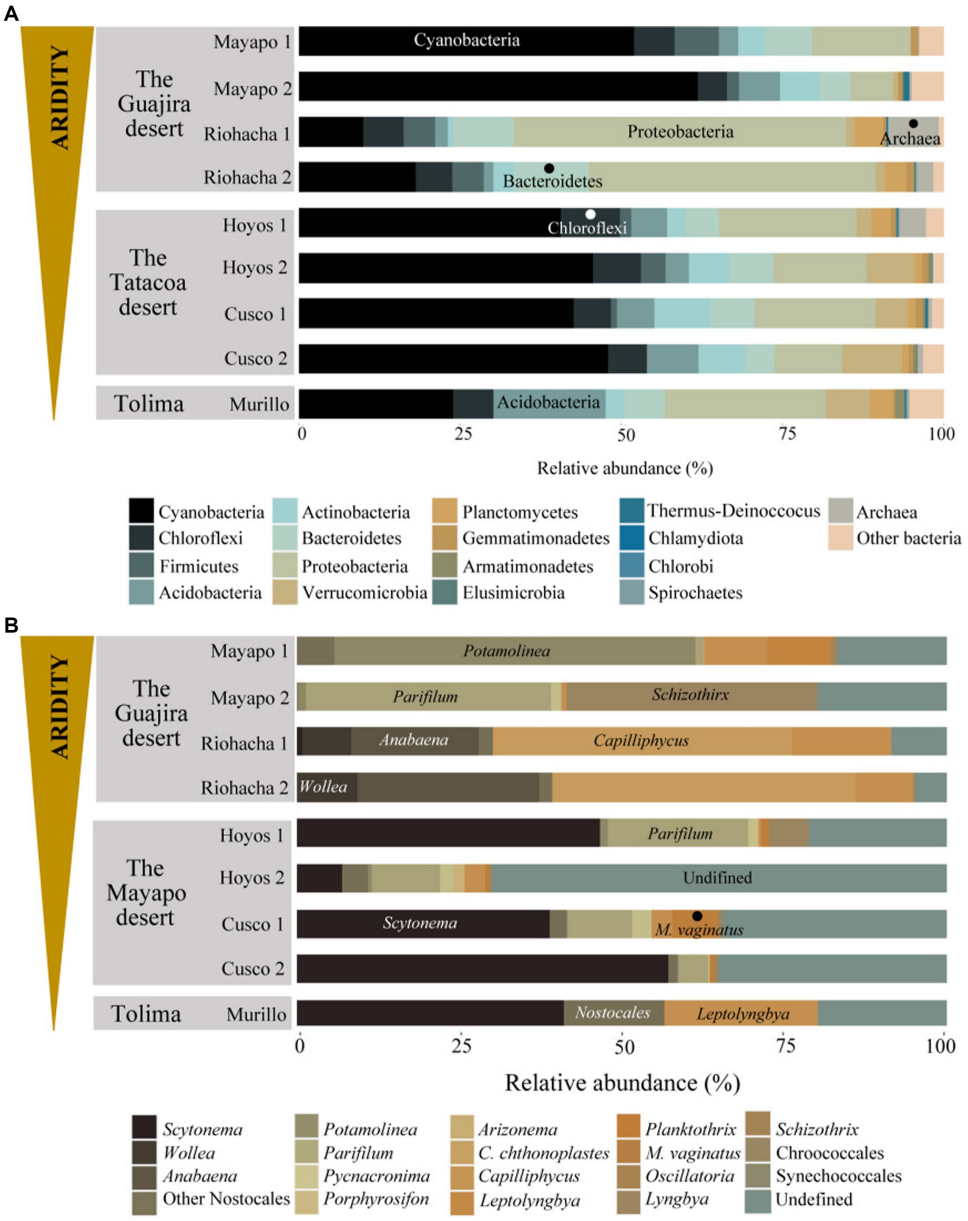
Microbial community structure of Colombian biocrusts. **(A)** Bacterial/Archaea, and **(B)** cyanobacterial communities. Relative abundance calculated by proportions of 16S rRNA gene sequence reads. Each plot represents average relative proportions of three independent determinations for each taxon resolved at the phylum level in panel **(A)** and minimally at the Genus level in panel **(B)**. Biocrusts are grouped by sampling locality within the studied climatic regions.

At all nine sites the Bacterial phyla Cyanobacteria, Proteobacteria, Actinobacteria, Acidobacteria, Bacteroidetes, Chloroflexi and Firmicutes accounted for more than 70% of the relatively more abundant community members (Figure 3A). Cyanobacteria was the most relatively abundant phylum (more than 40%) at all sites except in Riohacha (1 and 2) and Murillo where it accounted for about 14 and 24% (on average) of the microbial community, respectively. Cyanobacterial communities vary significantly between the three main sampled regions (the Guajira desert, the Tatacoa desert and Tolima; Permanova, pseudo-F: 6.95, *p* = 0.001, Supplementary Figure S2B). Within the Guajira desert region, communities from Mayapo 1 and 2 were different among each other (Pairwise permanova, pseudo-F: 12.27, *p* = 0.028, Figure 3B – Mayapo, Supplementary Figure S2B, and Supplementary Table S2). Both communities were dominated by filamentous cyanobacteria, in particular those from the Coleofasciculaceae family with ASV’s from *Potamolinea* spp. being overwhelmingly more relative abundant in communities from Mayapo1 and ASV’s from *Parifilum* spp. being more abundant in communities from Mayapo 2. The filamentous cyanobacterium *Schizothrix* sp. was also highly abundant in the later communities (Figure 3B). In contrast, within the same region (The Guajira desert), sites Riohacha1 and 2 were not different from each other (Pairwise permanova, pseudo-F: 1.12, *p* = 0.6, Figure 3B – Riohacha, Supplementary Figure S2, and Supplementary Table S2). In both communities the filamentous cyanobacteria *Capilliphycus* spp. was highly abundant (relative abundance: 39–53%). Likewise, cyanobacteria from the Nostocaceae family were commonly found (relative abundance: 27–43%), with *Wollea* spp., and *Anabaena* spp. being the most abundant representatives of the family within these biocrusts (Figure 3B – Riohacha 1 and 2). Cyanobacterial biocrusts from The Tatacoa desert region were not different among each other (Pairwise permanova results, Supplementary Table S2 and Supplementary Figure S2). Overall members of the Nostocaceae family were the most common cyanobacteria found in these communities. In particular, the nitrogen fixing cyanobacteria *Scytonema* spp. accounted for relative proportions ranging from 10 to 57% of the total cyanobacterial reads observed in communities from this climatic region (Figure 3B – Tatacoa desert). In addition, and despite our efforts to further curate cyanobacterial ASVs using Cydrasil3 and NCBI blastn (Altschul et al., 1990), the Tatacoa desert communities exhibited the highest proportions of unassigned ASV’s across the present study (up to 70%, in average), indicating that there is an unknown cyanobacterial diversity to discover in these areas. In light, such curation allowed us to further classify unassigned ASVs into major taxonomical groups (see Supplementary Figure S3). For example, out of ~70% of unassigned cyanobacterial ASVs from the site Los Hoyos 2 in The Tatacoa desert, ~ 42% were further classified as undefined members of the Coleofasciculaceae family, ~18% as undefined Oscillatoriales, while ~10% could not be placed within any major cyanobacterial group. Also, *M. vaginatus*, the most commonly reported cyanobacterium in biocrusts worldwide (Büdel, 2001) was only present in communities from this semi-arid region and in low abundances (ranging from 0.5 to 7%, in average), indicating that other filamentous cyanobacteria perform better in these particular environments. Nitrogen fixing cyanobacteria, mainly *Scytonema* spp., dominated cyanobacterial communities from Murillo, followed by the filamentous cyanobacterium *Leptolyngbya* spp. (Figure 3B – Murillo). These communities also displayed the highest number of reads assigned to Chloroplasts, with sequences falling mostly within the Chlorophyta division in relative proportions ranging from 31–66% (Supplementary Figure S4-Chloroplasts reads were removed from the complete dataset before diversity and ordination analysis were performed, see methods). Furthermore, the primer set we utilized enabled us to detect broad trends in archaeal community biodiversity (as they also binds to archaeal 16S rRNA genes). Euryarchaeota sequences were found exclusively in communities from the driest region, the Guajira desert, with proportions as high as 11% in Riohacha 1 and 2 (Table 2). By conducting further blasting, we were able to classify these microbial sequences as Halobacteria in the Halobacteriacea family. Additionally, we also identified the presence of Thaumarchaeota sequences in all sampled regions (but not all locations) in relative low proportions (1% max, see Table 2).

**Table 2:** Relative proportions of archaeal sequences observed across Colombian biocrusts.

**Location**	**La Guajira**				**La Tatacoa**				**Tolima**	**Taxomomy/Family**
	**Riohacha**		**Mayapo**		**Los Hoyos**		**Cusco**		**Murillo**	
	**1**	**2**	**1**	**2**	**1**	**2**	**1**	**2**		
**Relative proportions of Archaea sequences (%)**	11.50	3.29	0	0	0	0	0	0	0	Halobacteriaceae
	0.18	0.04	0	0.40	0	1.01	0.02	0.62	1.24	Nitrososphaeraceae

Assignment of Family taxonomy was performed usting the blastn algorithm from NCBI.

#### 3.2.1 Relating Colombian biocrust microbiomes diversity with environmental variables

**Figure 4:**
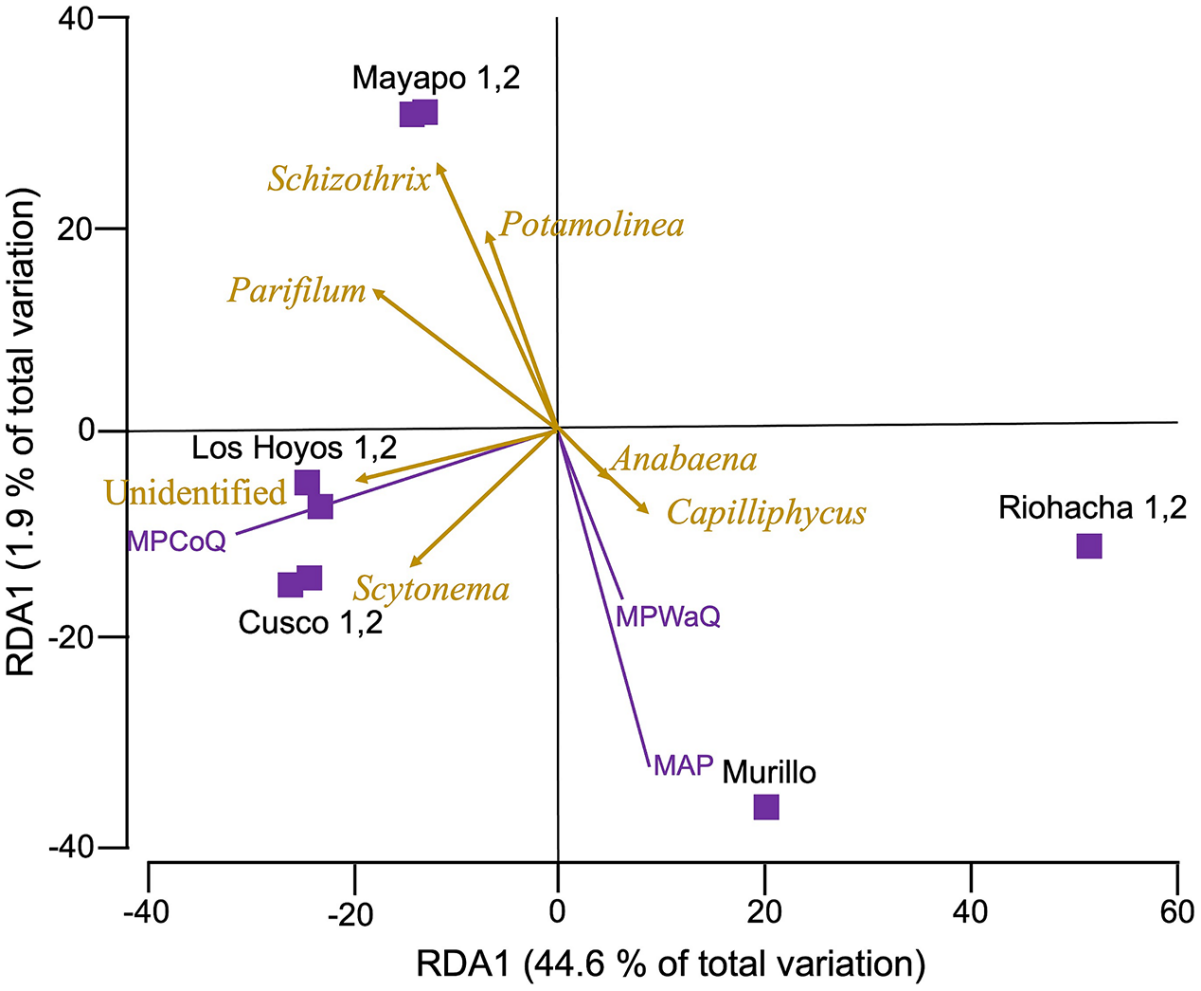
Redundance analysis (RDA) relating environmental variables with cyanobacterial diversity from biocrust communities in Colombia. Cyanobacterial community composition data was rarefied, and Hellinger transformed before a Bray Curtis dissimilarity matrix was obtained. Environmental variables were normalized before a Euclidean matrix was obtained. Vectors indicate those variables with the strongest correlation with microbial communities. Biocrusts are grouped by sampling location within major regions. Mayapo and Riohacha from the Guajira desert, Los Hoyos y Cusco from the Tatacoa desert and Murillo in the Tolima region. MPCoQ: annual mean precipitation during the coldest quarter of the year; MPWaQ: annual mean precipitation during the warmest quarter of the year; and AMP: annual mean precipitation.

Our RDA analysis (Figure 4) show that among the 10 environmental variables we tested (refer to methods and Supplementary Table S1), only three: mean annual precipitation during the coldest quarter of the year (MPCoQ), mean annual precipitation (MAP) and mean annual precipitation during the warmest quarter of the year (MPWaQ), exhibit a significant correlation with the diversity of biocrust cyanobacteria in the Colombian regions investigated in this study (sequential tests, *p* = 0.001 in all instances). Collectively, these three environmental variables explained 56% of the total observed variability. MPWaQ and MAP appear to influence the composition of biocrust cyanobacteria in communities located in Murillo. Moreover, MPCoQ emerged as the primary factor influencing cyanobacterial composition in communities found in the Tatacoa desert. Furthermore, it appears that the communities from The Guajira desert (Mayapo and Riohacha) are seeing a lesser degree of impact from these environmental variables associated with precipitation. The presence of *Schizothrix* spp., *Parifilum* spp. and *Potamolinea* spp. played a significant role in distinguishing the microbial composition of Mayapo 1 and 2 from those from the same arid Guajira desert (Riohacha 1 and 2). In contrast, the microbial composition of Riohacha 1 and 2 seem to be more defined by the presence of *Anabaena* spp. and *Capiliphycus* spp. *Scytonema* spp. and the high proportion of unidentified cyaobacterial sequences correlated positively with communities from the semi-arid Tatacoa desert. We ran the same analysis for the whole Bacterial community (Supplementary Figure S5) and found that similar to the cyanobacterial community MPCoQ and MAP explained about 41% of the observed variation (30.5 and 10.8%, respectively). However, it is less clear their particular influence toward local communities (sampling locations).

#### 3.2.2 Microbial community composition of biocrusts across the Americas

**Figure 5:**
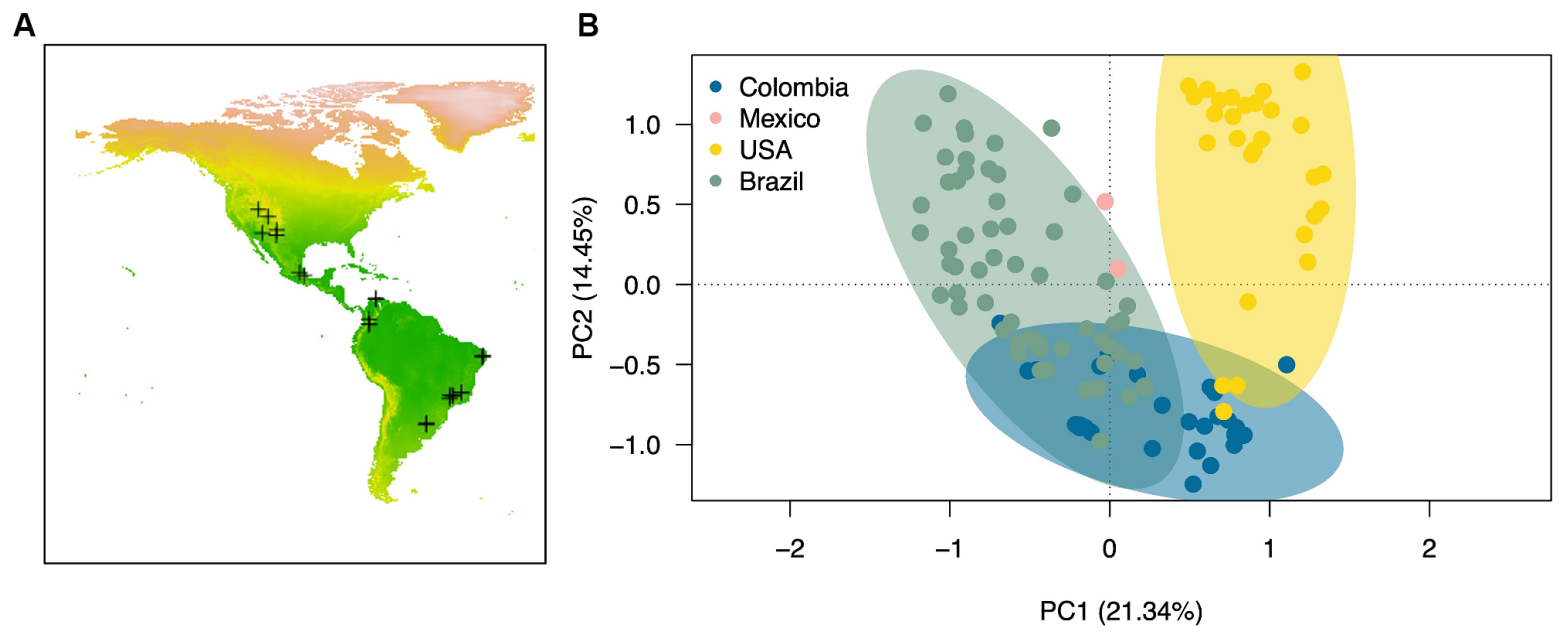
Biocrust cyanobacterial community composition across the American continent. **(A)** Map of the American continent showing the locations of the sampled locations included in the meta-analysis (see Supplementary Table S1 for further information), and **(B)** Principal component analysis based on Bray-Curtis pairwise distances calculated from the Hellinger transformed cyanobacterial sequencing data. Ellipses are drawn at a 95% confidence interval. Pairwise comparisons: *P* = <0.05 for all cases.

We compared the biocrust cyanobacterial community composition in the American continent by computing raw sequencing of 16Sr RNA genes data of 121 environmental biocrust samples across four countries (USA, Mexico, Colombia and Brazil) within the Americas (Figure 5A). Cyanobacterial communities differed significantly in composition across countries (Figure 5B; Permanova, Pseudo-*F* = 12.51, *p* = 0.001) exhibiting average dissimilarity percentages ranging from 67 to 80% for all groups-pairwise comparisons (Table 3; SIMPER analaysis; Groups were defined by country). Based on our SIMPER analysis the presence of *M. vaginatus* in biocrusts from USA drove in large proportion the differences observed between these communities and those from Mexico, Colombia and Brazil. In addition, the average proportion of the diazotrhophic cyanobacterium *Scytonema* largely drove the observed differences between Mexican and the rest of the analyzed American biocrusts communities. Furthermore, *Leptolyngbya* was the most important driver of the differences observed when comparing Brazialian biocrusts communities against Mexico, Colombia and USA. Finally, we did not observed an specific cyanobacterium driving the observed differences between Colombian biocrusts and the rest of the analyzed coutries indicating that proportionally there is a high Cyanobacterial compositional variability within Colombian biocrust communities (Table 3; Figure 3).

**Table 3:** Summary of SIMPER analysis results run on the Bray Curtis dissimilarity matrix.

**Group 1 and group 2**	**Average % dissimilarity**	**Major contributors**	**% Cyanobacteria contribution to dissimilarity**	**Average abundance (%)**	
				**Group 1**	**Group 2**
USA and Brazil	80	*M. vaginatus Leptolyngbya*	19.51 7.41	96.65 21.79	0.53 37.54
Colombia and USA	78	*M. vaginatus* Coleofasciculaceae	22.29 28.6	2.27 10.22	96.65 45.97
Mexico and Brazil	75	*Leptolyngbya* *Scytonema*	11.98 11.86	1.22 36.29	37.54 13.56
Colombia and Brazil	73	*Leptolyngbya* Unassigned	11.68 8.11	13.78 7.03	37.54 25.22
Mexico and USA	70	*M. vaginatus* *Scytonema*	22.93 10.16	8.63 36.29	96.65 15.39
Colombia and Mexico	67	*Scytonema* *Leptolyngbya* *Parifilum*	15.37 8.12 7.53	14.82 13.78 14.82	36.29 1.22 0.00

The table shows the average dissimilarity between groups, the major cyanobacterial contributors, and their percentage contribution to the observed dissimilarity along with their relative abundance for each group. Groups were defined as all the environmental tallies included in the metanalysis and sorted by country of origin within the Americas.

We also ran an RDA analysis to relate the cyanobacterial composition from biocrusts communities across the Americas with temperature and precipitation. All biocrust communities, except those from cold desert locations in USA, showed a positive correlation with the tested environmental variables. The two statistical axes represented in Supplementary Figure S6 explain 26.4% of the total observed variation; however, none of the tested environmental variables affected preferentially any of the biocrust communities included in the analysis, suggesting that our dataset does not have sufficient resolution to resolve the observed spatial patterns displayed by the studied biocrust cyanobacterial communities.

## 4 Discussion

Here we characterized the microbial diversity of biocrust microbiomes in three climate regions (arid, semi-arid and dry sub-humid) in Colombia by sequencing the 16S rRNA genes from these soil communities. Although the presence of these cryptogamic assemblages has been reported (Tabeni et al., 2014; Knudsen and Flakus, 2016; Rengifo and Arana, 2017; Velasco et al., 2020) and forecasted (Rodríguez-Caballero et al., 2018) across South America, our results represent the first description for Colombia, filling a gap in the biogeographical distribution of biocrusts worldwide. Furthermore, the results presented here constitute the first available genomic characterization of the complete bacterial community associated with biocrust microbiomes in South America.

### 4.1 Moisture timing and delivery regulate biocrust community structure

In Colombia, moisture timing and delivery appear to have a significant impact on the regulation of biocrust community diversity, particularly for cyanobacteria. The richness and diversity of biocrust cyanobacteria communities are lower in dry and warm areas with limited precipitation compared to semi-arid and warm areas with moderate precipitation, as well as in dry sub-humid areas with heavier rainy seasons and more moderate temperatures. Our findings are consistent with a classic morphological study conducted on Chilean biocrusts across a climatic gradient, which revealed an increase in cyanobacteria diversity from arid to semi-arid locations (Samolov et al., 2020). The same study found a high diversity of Chlorophyta, Streptophyte, and Ochlorophyte in temperate-humid environments, which is comparable to what we found in communities from the dry-subhumid area of Murillo communities (Supplementyary Figure S5). In addition, a 16S rRNA genes based study from Australia also reported climatic effects on biocrust biodiversity, with an increase in cyanobacterial diversity from locations with warmer winters and summer rainfalls to locations with cool winters associated and higher rain. While climatic regions studied in Australian are not a perfect match with the ones from the current study, in both regions biocrust cyanobacterial diversity increased with precipitation.This overall trend agrees with reports on biocrusts communities worldwide (Weber et al., 2022).

Our primer choice also allowed us to broadly detect biodiversity trends in archaeal communities (since the used set of primers also bind to Archaeal 16S rRNA genes). We observed novel community structures, within the driest communities including Euryarchaeota Archaea. We also observed previously-reported trends in Thaumarchaeota, detecting their ubiquitous presence in relative low abundances (Soule et al., 2009; Marusenko et al., 2013). Sequences assigned to the Euryarchaeota phylum were exclusively found in communities from the driest locations of the Guajira desert, specifically in those from Riohacha 1 and 2 in proportions as high as 11% (Table 2). Further blastn allowed us to classify these microbial sequences as members of the Halobacteria, a group of archaea, usually found in aquatic environments that need extremely high salt concentrations to grow and to survive (Ng et al., 2000). Beside saline environments, archaeal sequences matching at a 100% similary the ones in this study have been also recorded in agricultural soils and in the root of grasses sucha as *Ruppia* spp. and *Phragmites* spp. (according to blanstn performed on the NCBI website, Altschul et al., 1990). Previous studies targeting Archaea in biocrusts have only reported the presense of organisms in the Thaumarchaeota phylum as community members (Soule et al., 2009; Marusenko et al., 2013). It is plausible we observed this relative high proportion of Halobateria (Euryarchaeota) exclusively associated with biocrusts from the locales Riohacha 1 and 2 given they were collected in the shoreline, meters away from the Caribbean Sea. If this is the case, the overall decrease in Archaeal richness and diversity with increased precipitation we observed here may be unique to this dataset, indicating that presence of Halobacteria in biocrusts may be restricted to locations with high salinity content. A holistic study targeting Archaea in biocrusts across climatic gradients will need to take place to confirm or refute our findings, but most importantly to uncover the distribution, niche preferences and understudied role of members of this phylum in biocrust communities. Moreover, and in agreement with Soule et al. (2009) and Marusenko et al. (2013) sequences from the Thaumarchaeota phylum were obiquitous to biocrust communities from all the studied regions, in relative proportions that did not exceed 1%. Through blastn we assigned these sequences to ammonia oxidizing archaea (AOA; in the Nitrososphaeraceae family, see Table 2), which have been previously reported as potentially important biogeochemial agents in the cycling of N within biocrusts communities as ammonia oxidizers (Marusenko et al., 2013), particularly in warmer and drier dryland soils where they tend to replace ammonia oxidizing bacteria.

### 4.2 Pioneer and diazotrophic secondary colonizer cyanobacteria have a distinct structure in Colombian biocrusts

Colombian biocrusts are phylogenetically similar to each other at the phyla level (Figure 3A and Supplementary Figure S2A). They exhibited a bacterial structure that in composition and relative proportions resembles the arrangement of biocrusts worldwide with phyla as Cyanobacteria, Proteobacteria, Chloroflexi, Firmicutes, Acidobacteria, Actinobacteria and Bacteroidetes being the most important components of the communities [i.e., North America (Garcia-Pichel et al., 2013; Couradeau et al., 2016; Fernandes et al., 2018; Becerra-Absalón et al., 2019; Giraldo-Silva et al., 2019), Europe (Williams et al., 2016; Muñoz-Martín et al., 2018), Australia (Moreira-Grez et al., 2019; Chilton et al., 2022), and Asia (Zhang et al., 2016)].

As expected, Cyanobacteria were the most important community members in Colombian biocrusts (Figure 3). We observed that changes in the biocrust cyanobacterial structure were driven by a combinations of moisture and heat. Overall, the driest locations (Mayapo 1 and 2 in the Guajira desert) were mostly dominated by motile, dessication tolerant, non-nitrogen fixing filamentous cyanobacteria forming what is known in biocrust ecological succession as early or light biocrusts. As precipitation increased, changes in the biocrusts cyanobacterial structure match a progress in ecological succession and nonmotile, nitrogen-fixing cyanobacteria became more abundant (Figure 3), forming what is known as late successional or dark biocrusts. Among filamentous cyanobacteria, a high proportion of sequences across climatic regions were assigned to different members of the newly described Coleofasciculaceae family (Fernandes et al., 2021). Prior to the family’s description in 2021 all of these cyanobacteria were ascribed globally across biocrust surveys into a black box called *Microcoleus steenstrupii* (or *M. steenstrupii* complex), resulting in a loss of global diversity infomation and clearly representing an obstacle to understanding biocrusts local dynamics. In Colombian biocrusts we detected ASVs matching six (see Figure 3B) of the 11 genera described in the family. The more conspicuous patterns we observed were a high proportion of the pioneer cyanobacteria *Potamolinea* spp., and *Parifilum* spp. in extreme dry locales, followed by their lost in dominance with increasing precipitation, with *Parifilum* spp. showing restricted presence in dry-subhumid areas. Such a change in dominance among biocrust pioneer cyanobacteria with changing environmental conditions has also been observed in arid lands communities from USA where *M. vaginatus* is the architect organism in colder climates and loses dominance in warmer environemnts (Garcia-Pichel et al., 2013). The presense of the other Coleofasciculaceae family members detected in this study, *Picnacronima* spp., *Porphyrosyphon* spp., *Arizonema* spp. and *Coleofasciculus chthonolplastes*, was restricted to semi-arid locales, suggesting that extreme aridity and dry-subhumid climates are unsuitable environments for these cyanobacteria.

The current dataset, however, makes it impossible to determine whether the observed cyanobacterial distribution pattern is due to individual sensitivity to heat or water availability. This points to the need for laboratory investigations into the physiological responses of these cyanobacteria to heat and drought. These types of studies will be critical to understanding the ability of biocrust communities to deliver ecosystem services in a changing climate. These ecosystem services are regulated by local community dynamics, driven by the direct link between biocrusts species composition and the ecological properties and ecosystems services they render to local soils.

Other filamentous cyanobacteria found in high abundance in Colombian biocrusts were *Capyliphycullus* spp., *Leptolyngbya* spp., and *Schizothrix* spp. *Capyliphycullus* spp. was surprisingly found in high proportions associated to microbial communities from extreme arid environments that are also high in salt concetration due to their proximity to the Caribbean sea (Riohacha 1 and 2). *Capyliphycullus* spp., is a halophilic genus in the Microcolaceae family that can display desiccation tolerance (Berthold et al., 2022), a set of physiological traits that makes it suitable to colonize such environments. The presence of halotolerant-halophillic cyanoabacteria is not rare in biocrust communities (Bowker et al., 2016) neither in arid lands soils with high salt content (~18 ppm) (Beraldi-Campesi and Garcia-Pichel, 2011), where the marine cyanobacterium *Coleofasciculus chthonoplastes* is dominant. *Leptolyngbya* spp., and *Schizothrix* spp., also highly abundant in Colombian biocrusts, are common members of biocrusts communities (Garcia-Pichel et al., 2013; Muñoz-Martín et al., 2018; Becerra-Absalón et al., 2019; Giraldo-Silva et al., 2019; Machado-de-Lima et al., 2019), but rarely appeared as dominant until in 2019 *Leptolyngbya* was reported as the most common cyanobacterium in Brazilian biocrusts from semi-humid tropical regions (Machado-de-Lima et al., 2019). The relative proportions exhibited by *Leptolyngbya* in Brazilian communities closely mirror those reported in USA communities for the pioneer dominant species *M. vaginatus* (Fernandes et al., 2018; Giraldo-Silva et al., 2019), considered as one of the most successful cyanobacteria in biocrust communities worldwide (Büdel, 2001). This is in agreement with reports from Mexico (Becerra-Absalón et al., 2019), and this same study, where *M. vaginatus* was almost absent in the surveyed communities. In Colombian biocrusts, it was only found in the semi-arid region of the Tatacoa desert in low abundance. This supports the notion that biocrust microbial players are highly selected by regional environmental conditions, and the need to further characterize local diversity given the difference in microbial actors across climatic regions.

Colombian biocrust soils displayed a distinctive nitrogen fixing and secondary colonizer cyanobacterial structure that differed across climatic regions. *Wollea* spp. and *Anabaena* spp. showed dominance under extremely arid and high salt content environments (Riohacha), while *Scytonema* spp. was highly abundant in semi-arid (Tatacoa desert) and in dry-subhumid (Murillo) regions. Neither *Wollea* spp. nor *Anabaena* spp., have been reported as important biocrusts community members in the Americas (USA, Mexico, Brazil). Instead, *Nostoc* spp., and *Tolypothrix* spp., which were not detected in Colombian biocrusts, are considered an abundant taxa in colder climates in North American arid lands (Becerra-Absalón et al., 2019; Giraldo-Silva et al., 2020), while *Scytonema* spp., gains dominance in warmer regions. *Scytonema* spp., however, was the dominant diazotrhophic cyanobacteria in cold dry-subhumid regions in Colombian biocrusts, which contrasts with previously shown global trends for this cyanobacterium in biocrusts (Giraldo-Silva et al., 2020). Nitrogen fixing cyanobacteria usually come second in biocrusts ecological succession gaining dominance over diazo-heterotrophic bacteria in the cyanosphere of pioneer filamentous cyanobacteria (Couradeau et al., 2019), and just after pioneer organisms have colonized and stabilized bare soils. Due to the capability of incorporating dinitrogen from the atmosphere into the soils they cover, these cyanobacteria represent the main biological source of nitrogen inputs (Dodds, 1989) not only for biocrust communities but also to adjacent nutrient poor soils (Beraldi-Campesi et al., 2009). They also act as a shield, protecting the biocrust community from UV radiation by producing accessory pigments suchs as Scytonemin (Garcia-Pichel and Castenholz, 1991), mycosporine-like amio acids (Shibata, 1969) and carotenoids (Paerl, 1984) in response to high photon exposure. The expression of such pigmented compounds prevents cellular damage by photooxidation over long preriods of light exposure when organismas are not biologically active due to intermitten dessication periods, and during nutrient-limited conditions (Garcia-Pichel and Castenholz, 1991), which are typical environmental stressors in our sampling locations.

Furthermore, a high proportion of ASV’s in Colombian biocrusts found distant phylogenetic relatives in common global bacterial databases, resulting in a high proportion of unassigned diversity. A similar trend has emerged from recent studies in Brazil (Machado-de-Lima et al., 2019) and Australia (Chilton et al., 2022) suggesting that communities from understudied areas contain a significant diversity component that has yet to be identified and that could even harbor endemic species. This hypothesized hidden diversity, together with the observed biocrust cyanobacterial structure and their distribution across the studied climatic gradients in Colombian communities, highlights the need for new genetic surveys. Moreover, it is important to conduct these surveys alongside microscopic studies, the isolation and characterization of local key species, and the examination of their physiological and ecological traits in order to gain a comprehensive understanding of regional dynamics. This is necessary because the majority of global biocrust frameworks have been developed based on arid communities in the United States.

### 4.3 Seasonal precipitation drives growing season in Colombian biocrusts

**Figure 6:**
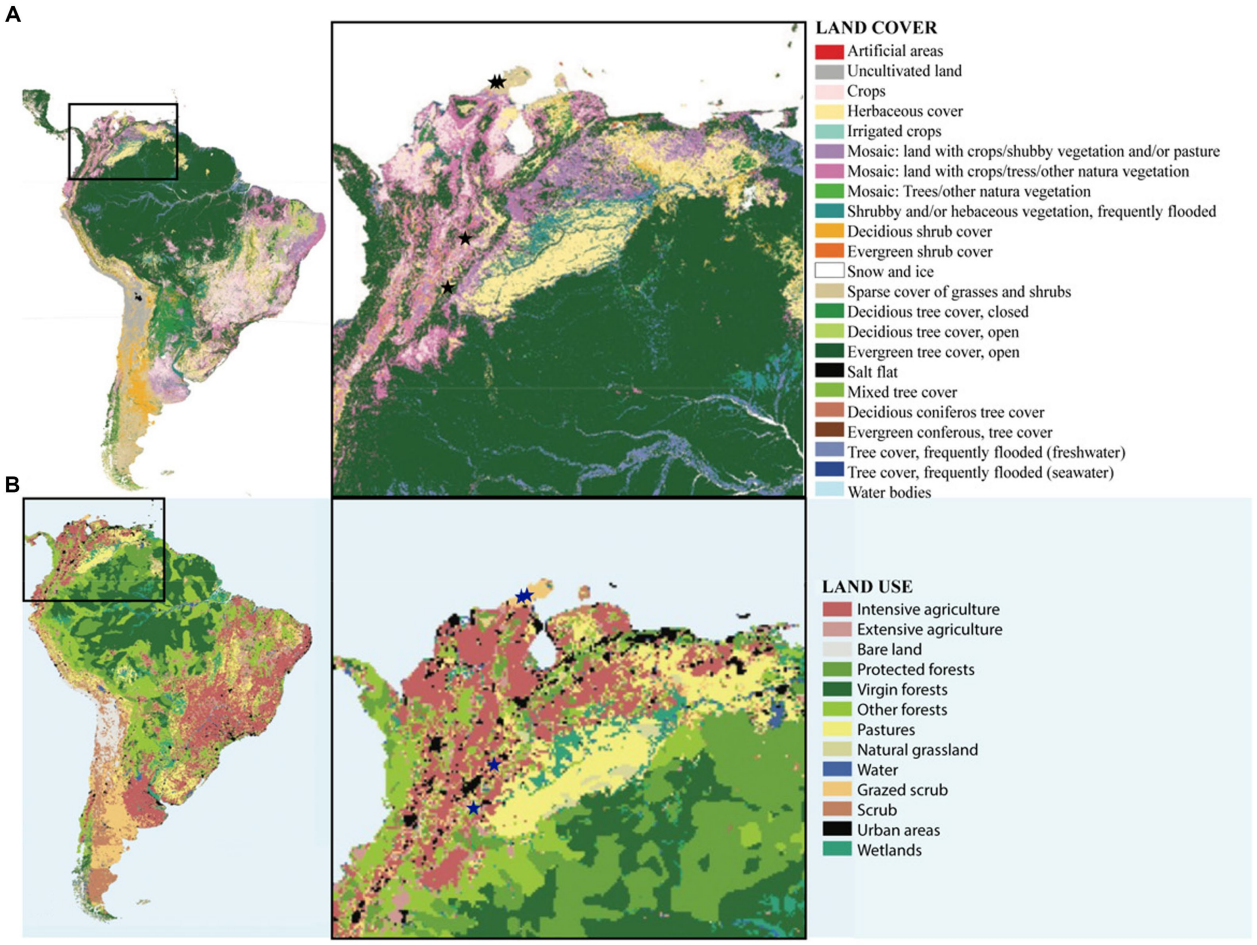
Maps of vegetation cover and land use in Colombia. **(A)** Map of type of vegetation cover showing the locations of the sampled sites, and **(B)** Map of type of land use showing the locations of the sampled sites.

According to our RDA analysis precipitation during the coldest and the warmest quarter of the year (growing season) were the primary forces driving the observed variation in bacterial and cyanobacterial composition in Colombian biocrusts from semi-arid and dry sub-tropical regions. Aridity (temperature and precipitation) has been found to drive variations in species composition in arid lands in USA (Garcia-Pichel et al., 2013; Fernandes et al., 2018), Australia (Chilton et al., 2022) and in Mediterranean biocrusts (Muñoz-Martín et al., 2018). Even though precipitation and temperature have been shown to significantly affect biocrust diversity, in this study, the tested environmental variables accounted for roughly 50% of the observed variability, indicating that in these enviroments, not only environmental but other factors such as soil pH, soil moisture, edaphic origin are also important microbial drivers. Linking metrics related to soil physicochemical characteristics will be critical for gaining a thorough understanding of the mechanisms determining structure and coverage in these biocrust communities. It is possible that a larger scale, climatic variables play an important role; however, it has been shown that soil parameters (i.e., soil temperature, edaphic origin and pH) act as the most important diversity drivers at the local scale (Cano-Díaz et al., 2018; Machado de Lima et al., 2021). Furthermore, biocrust microbial configurations are impacted by patterns of local land use and disturbances, which in turn reverse ecological succession and lead to early succession communities (Ferrenber et al., 2015). Early biological succession communities consisting primarily of pioneer motile filamentous cyanbacteria (Figure 2B) were observed in the dry localities Mayapo 1 and 2 in the Guajira desert, where goat grazing is severe (Figure 6B). The presence of secondary colonizers (no motile nitrogen fixing cyanobacteria, Figure 2B) in Riohacha 1 and 2, also in the arid Guajira desert, suggests that local aridity allows secondary colonozers to thrive. This indicates that local grazing circumstances may hinder Mayapo’s communities from advancing in their natural succession, and that local microbial recruitment in the area is essential in keeping these communities viable in spite of the significant grazing pressure they face. Conversely, communities from the semi-arid Tatacoa desert, which is a national park and protected region with sparce cover of grasses and shurbs (Figure 6A) display communities where secondary cyanobacteria are prevalent and exist in high relative quantities. In Tolima, where crops are in mosaic with natural vegetation, and not grazing pressure is present, biocrust communities are conspicuous (Figure 1E) and characterize by a large proportion of secondary colonizers.

Furthermore, while our RDA analysis does not reveal specific microbial preferences to a given climatic setting, it does allow us to infer that in the semi-arid Tatacoa desert where the MAT is >27°C and summers are hot, the majority of the microbial growth occurs during the coldest months of the year, whereas in Murillo, where the MAT is 11°C, the highest peak in growth may be associated with the warmest months of the year. In the Guajira desert, however, where aridity conditions are more extreme, microbial growth may be restricted to climatic events where temperature and water availability are optimal for allowing biological activity to exceed the net compensation point required to replaced the respired carbon (Johnson et al., 2012), preventing communities from these locales to progress in their ecological succession as evidenced by our observation and microbial surveys (Figure 3).

Biocrust communities worldwide are expected to be impacted by climate change and land use, resulting in changes in dominance and/or replacement of species, and possibly in a redistribution of these soil microbiomes according to their physiological capabilities to adapt to new extreme environments. Our investigation represents the first diversity report of these soil microbiomes in Colombia and South America, one of the most understudied regions worldwide. Uncovering soil microbiomes’ biodiversity and linking their microbial compositional configurations across biomes with organisms’ physiology is key to understanding the effects of environmental factors on their function and thus the overall ecological roles of these soil microbiomes. In addition, and given the global extension of drylands, and the relevance they have for human population in terms of ecology and socioeconomical activities, increasing our knowledge on the biodiversity, structure and functioning of their soil microbiomes is also central to better design management and restoration strategies based on land use conditions and their forecasted responses to current and future climate change.

## Data availability statement

The datasets presented in this study can be found in online repositories. The names of the repository/repositories and accession number(s) can be found at: NCBI: PRJNA1007369.

## Author contributions

AG-S and CM conceived the research, edited and contributed to the discussion, and finalized the manuscript. AG-S sampled the biocrusts, performed the laboratory research, processed and analysed the data, and wrote the manuscript. All authors listed have made a substantial, direct, and intellectual contribution to the work and approved it for publication.

## Footnotes

^1^ https://www.cydrasil.org

^2^ https://ncbi.github.io/cxx-toolkit/

^3^ http://www.worldclim.org
