# Supplementary material for: Environmental conditions play a key role in controlling the composition and diversity of Colombian biocrust microbiomes
Source: Front Microbiol. 2024 Apr 25;15:1236554. doi: 10.3389/fmicb.2024.1236554 (PMC11081033; doi:10.3389/fmicb.2024.1236554)
Supplement: Supplementary file 1 [file Data_Sheet_1.pdf]

Supplementary Material

Environmental conditions play a key role in controlling the composition and diversity of Colombian biocrust microbiomes.

Ana Giraldo-Silva[1,2*] and Caroline A. Masiello[2]

[1] Ecology and Environemntal research group and Institute for Multidisciplinary Research in Applied Biology (IMAB), Department of Science, Public University of Navarre (UPNA), Campus Arrosadia, 31006 Pamplona, Spain.

[2] Department of Earth, Environmental and Planetary Sciences, Rice University, Houston, TX, USA

*** Correspondence:**
Corresponding Author

ana.giraldo@unavarra.es

1 Supplementary Figures

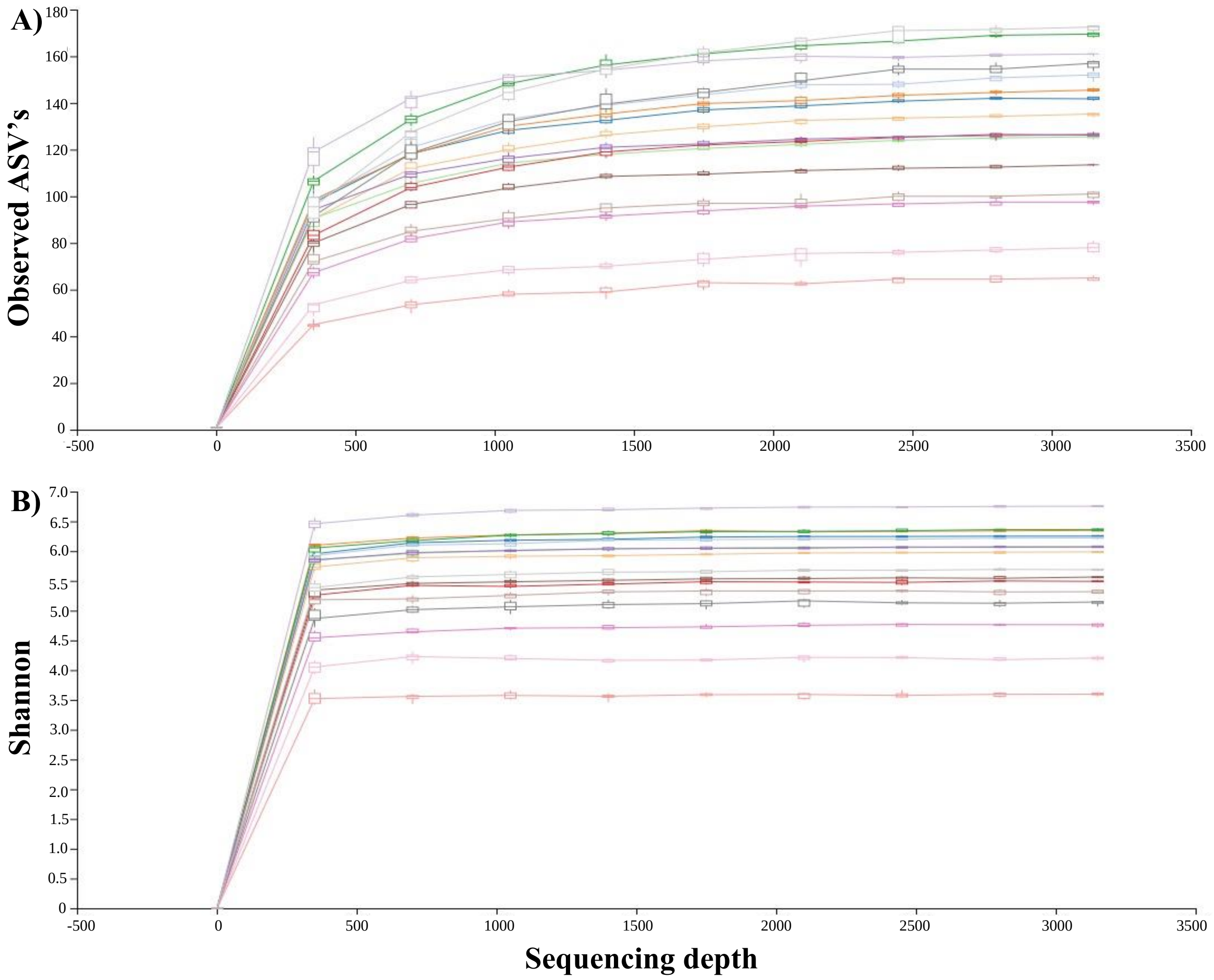

Supplementary Figure 1. Rarefactions curves for the whole dataset showing sequencing depth versus A) observed ASV's and B) Shannon's diversity index for 28 samples from nine locations. ASV's: amplicon sequence variants. Rarefaction curves showed that to a sampling depth of 3150 all samples reached a plateau, suggesting that collecting sequences beyond this sampling depth would not be likely to result in the addition of new features. Note: the minimal feature count for bacteria was 3150, while the maximum feature count was 8273. The lowest feature count for cyanobacteria was 304, and the maximum feature count was 5159. In both cases rarefaction curves reached a plateau at the rarefaction.

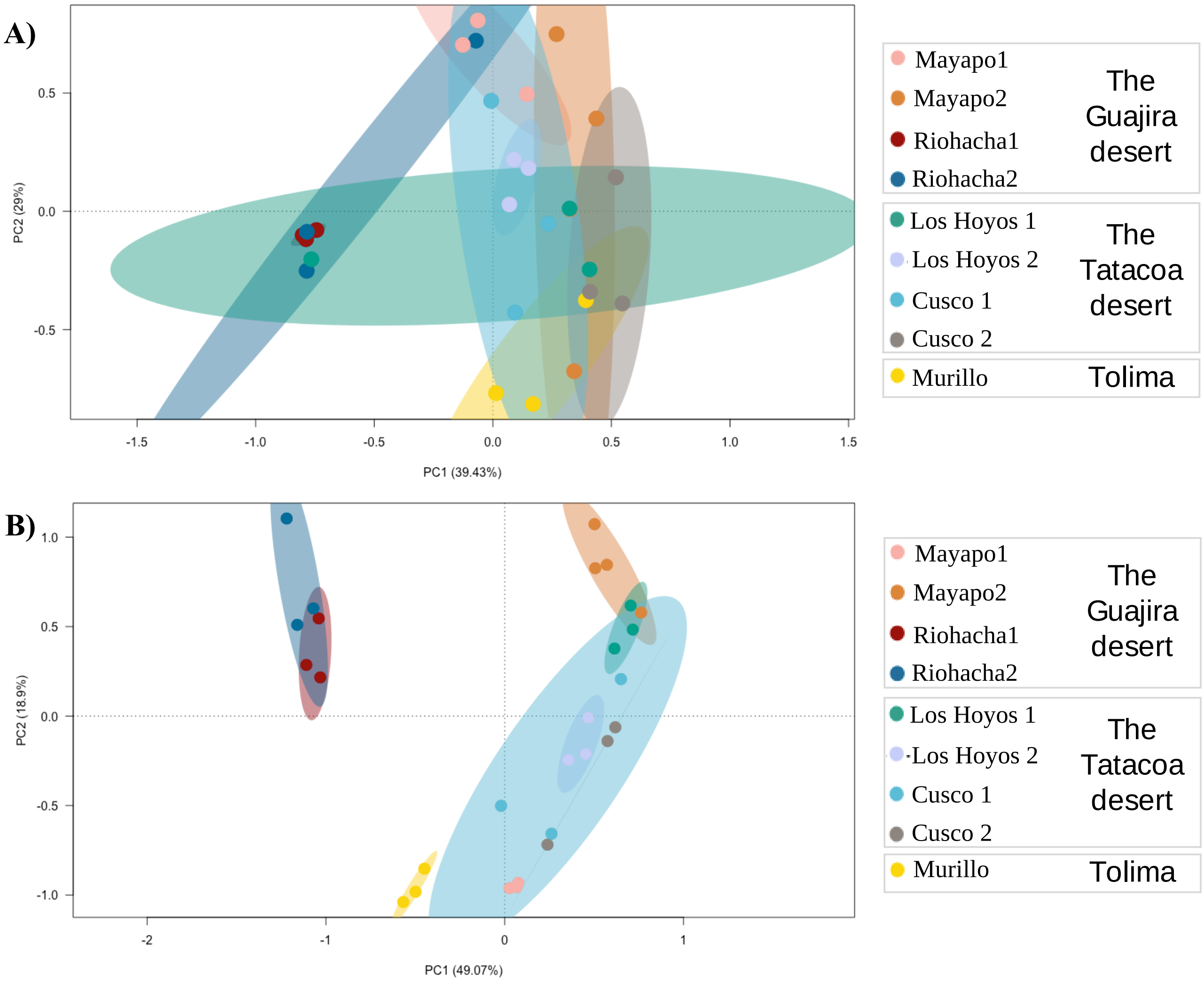

Figure S2. Principal component analysis (PCoA) for A) Bacterial and B) Cyanobacterial communities within Colombia. Plots were constructed based on Bray-Curtis pairwise distances calculated from the Hellinger transformed sequencing data. Biocrust communities are displayed by sampling location. Ellipses are draw at a 95 % confidence interval.

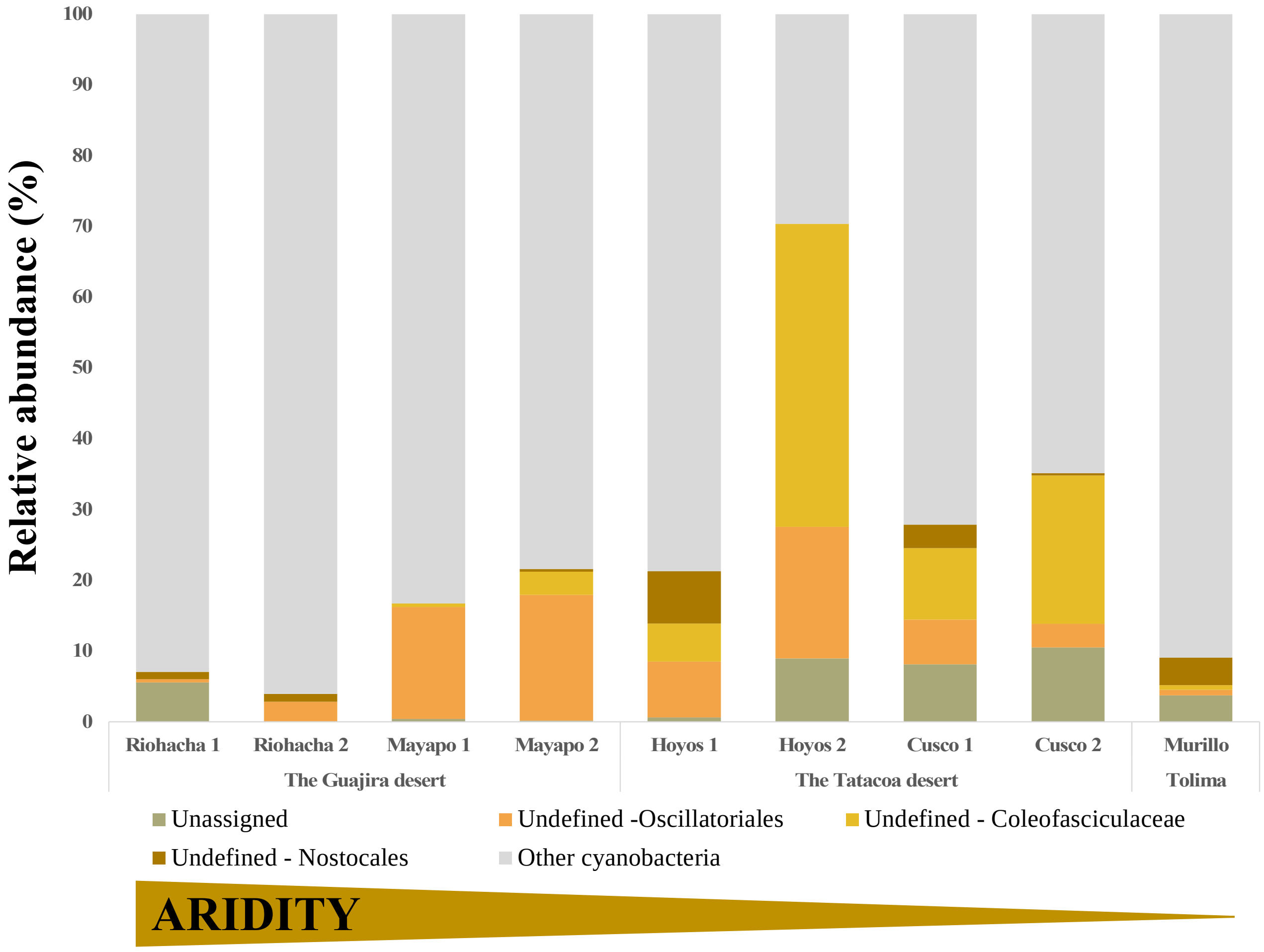

Figure S3. Structure of unassigned cyanobacterial ASV's from Colombian biocrusts tallies. Further classification of unassigned ASV's into major cyanobacterial groups based on Cydrasil3 and blastn. Biocrust communities are displayed by sampling location within the studied climatic regions.

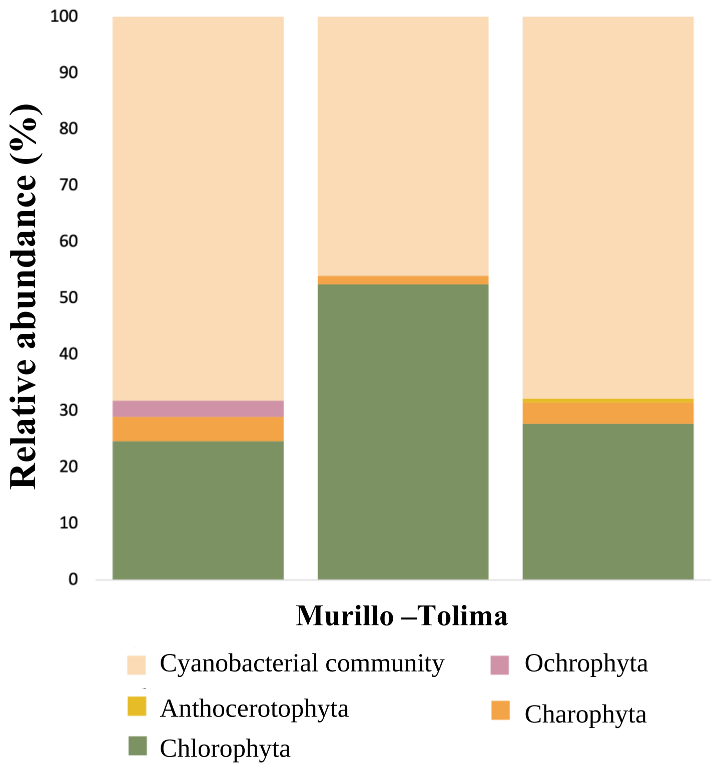

Figure S4. Structure of ASV's assigned as Chloroplasts in Colombian biocrusts from Murillo (Tolima). Chloroplasts reads were removed from the dataset before performing diversity and ordination analysis and are shown here to illustrate that communities from Murillo, a dry sub-humid location, displayed a high proportion of unicellular microalgae reads.

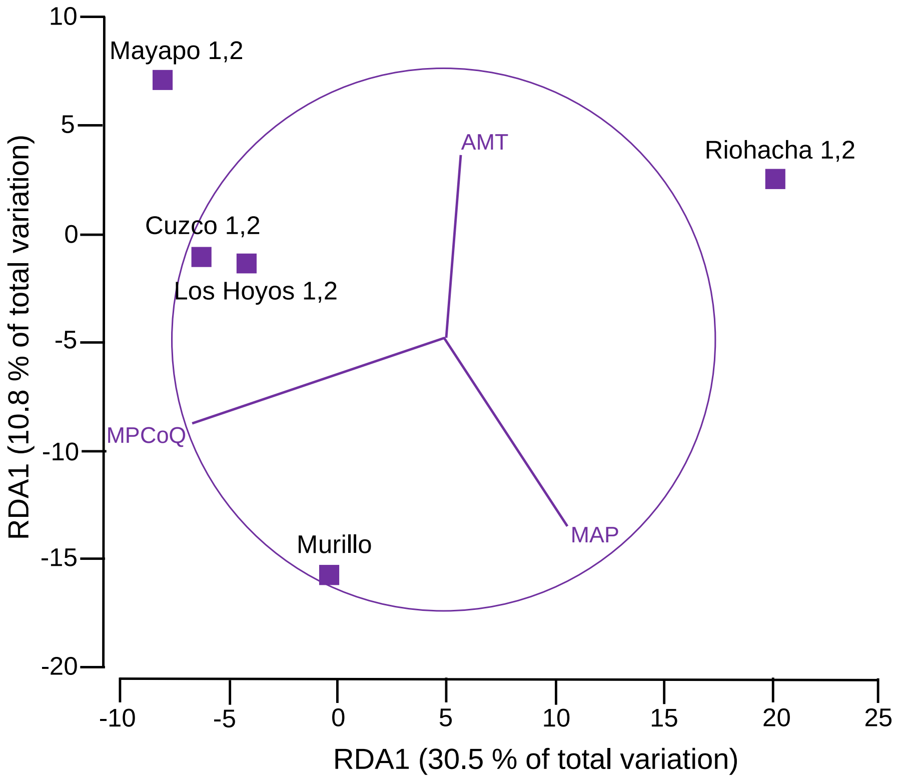

Figure S5. Redundance analysis (RDA) relating environmental variables with Bacteria/Archaeal community composition (all phyla) from biocrust communities in Colombia. Bacterial community composition data was rarefied, and Hellinger transformed before a Bray Curtis dissimilarity matrix was obtained. Environmental variables were normalized before a Euclidean matrix was obtained. Vectors indicate those variables with the strongest correlation with bacterial communities. Biocrusts are grouped by sampling location within major regions. Mayapo and Riohacha in the Guajira desert, Los Hoyos and Cusco in the Tatacoa desert and Murillo in the Tolima region. **MPCoQ***:* annual mean precipitation during the coldest quarter of the year, **MAP**: mean annual precipitation, and **MAT**: mean annual temperature.

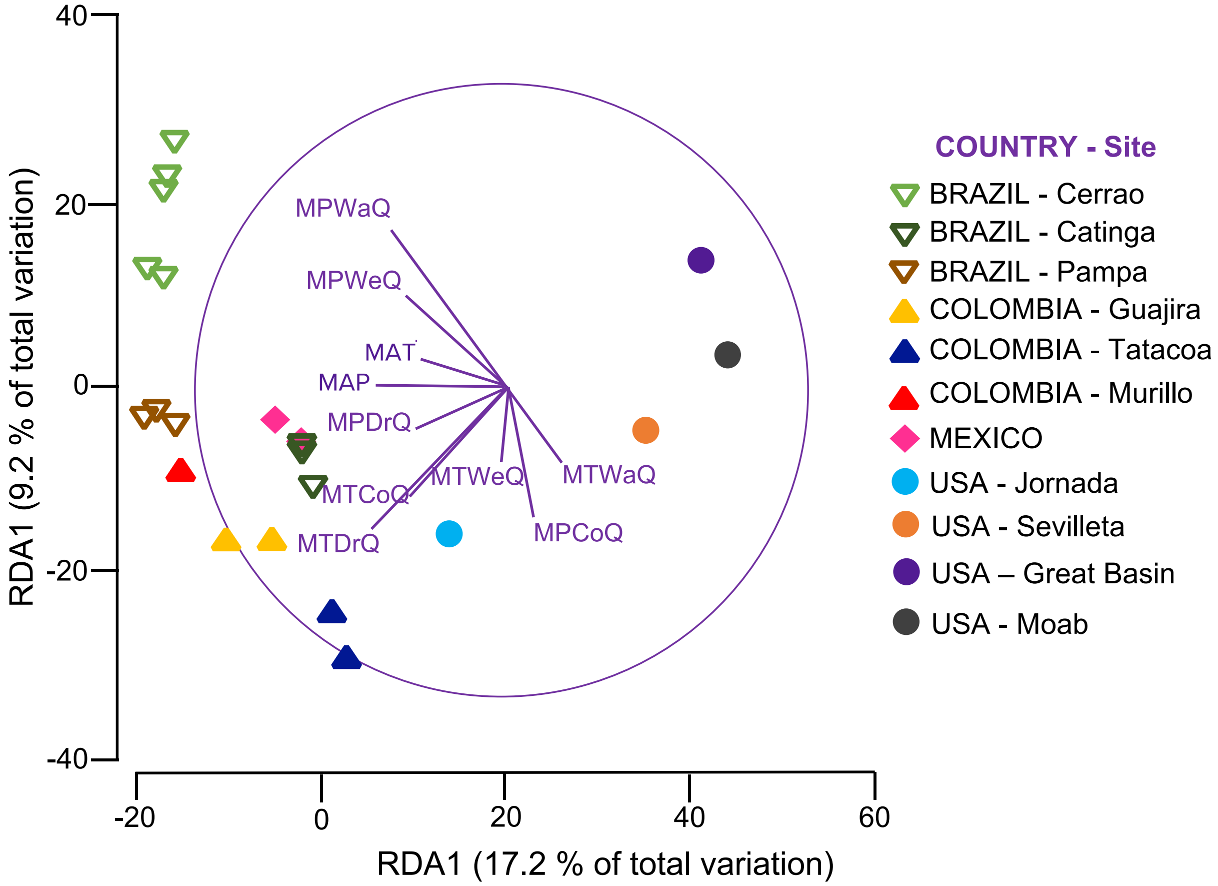

Figure S6. Redundance analysis (RDA) relating environmental variables of cyanobacterial community composition from biocrust communities in the American continent. Cyanobacterial community composition data was rarefied, and Hellinger transformed before a Bray Curtis dissimilarity matrix was obtained. Environmental variables were normalized before a Euclidean matrix was obtained. Vectors indicate those variables with the strongest correlation with microbial communities. Biocrusts are grouped by sampling regions within each country. For full name of environmental variables see table S1.

2 Supplementary Tables

Supplementary Table S1. Environmental biocrust surveys included in meta-analysis of biocrust communities from the Americas. Data corresponds to 121 locations across four countries from the American continent. Raw sequences were download from bacterial 16S rRNA sequencing available publicly (see references). Environmental data was downloaded from WorldClim (see methods). MAT: annual mean temperature, TWeQ: Mean annual temperature during the wettest quarter of the year, TDrQ: Mean annual temperature during the driest quarter of the year, TWaQ: Mean annual temperature during the warmest quarter of the year TCoQ: Mean annual temperature during the coldest quarter of the year, MAP: annual mean precipitation, PWeQ: Mean annual precipitation during the wettest quarter of the year, PDrQ: Mean annual precipitation during the driest quarter of the year, PWaQ: Mean annual precipitation during the warmest quarter of the year, and PCoQ: Mean annual precipitation during the coldest quarter of the year.

Original location descriptor	Latitude	Longitude	MAT	Mean TWeQ	Mean TDrQ	Mean TWaQ	Mean TCoQ	MAP	Mean PWeQ	Mean PDrQ	Mean PWaQ	Mean PCoQ	Country/Reference
F1	32.59194	-106.85286	14.8	238	143	247	50	257	140	22	120	40	USA/ (Nelson & Giraldo-Silva et al., 2022)
F2	32.59194	-106.85286	14.8	238	143	247	50	257	140	22	120	40	
F3	32.59194	-106.85286	14.8	238	143	247	50	257	140	22	120	40	
F4	32.59194	-106.85286	14.8	238	143	247	50	257	140	22	120	40	
F5	32.59194	-106.85286	14.8	238	143	247	50	257	140	22	120	40	
HSNA	41.104211	-113.008204	10.3	144	-16	227	-16	262	88	52	62	52	USA/ (Giraldo-Silva et al., 2019)
HSNB	41.104211	-113.008204	10.3	144	-16	227	-16	262	88	52	62	52	
HSNC	41.104211	-113.008204	10.3	144	-16	227	-16	262	88	52	62	52	
MRME	34.33776	-106.72821	12.6	219	45	228	25	250	127	30	108	34	USA/ (Fernandes et al., 2018)
MRME	34.33776	-106.72821	12.6	219	45	228	25	250	127	30	108	34	
MRME	34.33776	-106.72821	12.6	219	45	228	25	250	127	30	108	34	
MRME	34.33776	-106.72821	12.6	219	45	228	25	250	127	30	108	34	
MRME	34.33776	-106.72821	12.6	219	45	228	25	250	127	30	108	34	
M1A	38.7149722	-109.692944	11.9	189	-6	243	-6	229	73	46	56	46	USA/ (Couradeau et al., 2016)
M1B	38.7149722	-109.692944	11.9	189	-6	243	-6	229	73	46	56	46	
M1C	38.7149722	-109.692944	11.9	189	-6	243	-6	229	73	46	56	46	
M2A	38.7149722	-109.692944	11.9	189	-6	243	-6	229	73	46	56	46	
M2B	38.7149722	-109.692944	11.9	189	-6	243	-6	229	73	46	56	46	
M2C	38.7149722	-109.692944	11.9	189	-6	243	-6	229	73	46	56	46	
M3A	38.7149722	-109.692944	11.9	189	-6	243	-6	229	73	46	56	46	

M3B	38.7149722	-109.692944	11.9	189	-6	243	-6	229	73	46	56	46	
M3C	38.7149722	-109.692944	11.9	189	-6	243	-6	229	73	46	56	46	
M4A	38.7149722	-109.692944	11.9	189	-6	243	-6	229	73	46	56	46	
M4B	38.7149722	-109.692944	11.9	189	-6	243	-6	229	73	46	56	46	
M5C	38.7149722	-109.692944	11.9	189	-6	243	-6	229	73	46	56	46	
M5A	38.7149722	-109.692944	11.9	189	-6	243	-6	229	73	46	56	46	
M5B	38.7149722	-109.692944	11.9	189	-6	243	-6	229	73	46	56	46	
M5C	38.7149722	-109.692944	11.9	189	-6	243	-6	229	73	46	56	46	
Actopan	20.2674722	-98.9161111	15.5	165	130	178	128	672	327	33	221	43	MEXICO/ (Becerra-Absalón et al., 2019)
Atexcac	19.3369444	-97.3552778	14.5	162	120	164	120	393	174	21	160	23	
Riohacha 1	11.551892	-72.9115056	28.4	282	274	296	272	578	350	6	88	29	COLOMBIA/ This study
Riohacha 1	11.551892	-72.9115056	28.4	282	274	296	272	578	350	6	88	29	
Riohacha 1	11.551892	-72.9115056	28.4	282	274	296	272	578	350	6	88	29	
Riohacha 2	11.551892	-72.9115056	28.4	282	274	296	272	578	350	6	88	29	
Riohacha 2	11.551892	-72.9115056	28.4	282	274	296	272	578	350	6	88	29	
Riohacha 2	11.551892	-72.9115056	28.4	282	274	296	272	578	350	6	88	29	
Mayapo 1	11.6518611	-72.7826833	28.5	284	275	297	274	501	331	4	64	25	
Mayapo 1	11.6518611	-72.7826833	28.5	284	275	297	274	501	331	4	64	25	
Mayapo 1	11.6518611	-72.7826833	28.5	284	275	297	274	501	331	4	64	25	
Mayapo 2	11.6569222	-72.7861472	28.5	284	275	297	274	501	331	4	64	25	
Mayapo 2	11.6569222	-72.7861472	28.5	284	275	297	274	501	331	4	64	25	
Mayapo 2	11.6569222	-72.7861472	28.5	284	275	297	274	501	331	4	64	25	
Mayapo 2	11.6569222	-72.7861472	28.5	284	275	297	274	501	331	4	64	25	
Murillo	4.8504779	-75.177785	11.4	111	113	116	111	2060	668	351	518	610	
Murillo	4.8504779	-75.177785	11.4	111	113	116	111	2060	668	351	518	610	
Murillo	4.8504779	-75.177785	11.4	111	113	116	111	2060	668	351	518	610	
Tatacoa-Hoyos-1	3.2308	-75.1694	27.5	267	278	281	267	1275	536	89	119	536	
Tatacoa-Hoyos-1	3.2308	-75.1694	27.5	267	278	281	267	1275	536	89	119	536	
Tatacoa-Hoyos-1	3.2308	-75.1694	27.5	267	278	281	267	1275	536	89	119	536	
Tatacoa-Hoyos-2	3.2308	-75.1694	27.5	267	278	281	267	1275	536	89	119	536	
Tatacoa-Hoyos-2	3.2308	-75.1694	27.5	267	278	281	267	1275	536	89	119	536	
Tatacoa-Hoyos-2	3.2308	-75.1694	27.5	267	278	281	267	1275	536	89	119	536	

Tatacoa-Cusco-1	3.2279722	-75.1394417	27.1	264	275	278	264	1216	510	91	117	510	
Tatacoa-Cusco-1	3.2279722	-75.1394417	27.1	264	275	278	264	1216	510	91	117	510	
Tatacoa-Cusco-1	3.2279722	-75.1394417	27.1	264	275	278	264	1216	510	91	117	510	
Tatacoa-Cusco-2	3.2279722	-75.1394417	27.1	264	275	278	264	1216	510	91	117	510	
Tatacoa-Cusco-2	3.2279722	-75.1394417	27.1	264	275	278	264	1216	510	91	117	510	
Tatacoa-Cusco-2	3.2279722	-75.1394417	27.1	264	275	278	264	1216	510	91	117	510	
Cipo	-19.33	-43.56	18.4	200	162	204	158	1581	848	35	632	54	BRAZIL/ (Machado de Lima et al., 2019)
Cipo	-19.33	-43.56	18.4	200	162	204	158	1581	848	35	632	54	
Cipo	-19.33	-43.56	18.4	200	162	204	158	1581	848	35	632	54	
Cipo	-19.33	-43.56	18.4	200	162	204	158	1581	848	35	632	54	
Cipo	-19.33	-43.56	18.4	200	162	204	158	1581	848	35	632	54	
Cipo	-19.33	-43.56	18.4	200	162	204	158	1581	848	35	632	54	
Cipo	-19.33	-43.56	18.4	200	162	204	158	1581	848	35	632	54	
Capao	-19.3333	-43.5666	18.4	200	162	204	158	1581	848	35	632	54	
Capao	-19.3333	-43.5666	18.4	200	162	204	158	1581	848	35	632	54	
Capao	-19.3333	-43.5666	18.4	200	162	204	158	1581	848	35	632	54	
Capao	-19.3333	-43.5666	18.4	200	162	204	158	1581	848	35	632	54	
Capao	-19.3333	-43.5666	18.4	200	162	204	158	1581	848	35	632	54	
Capao	-19.3333	-43.5666	18.4	200	162	204	158	1581	848	35	632	54	
Zagaia	-21.316666	-47.616666	21	228	182	228	180	1483	740	78	649	105	
Zagaia	-21.316666	-47.616666	21	228	182	228	180	1483	740	78	649	105	
Zagaia	-21.316666	-47.616666	21	228	182	228	180	1483	740	78	649	105	
Zagaia	-21.316666	-47.616666	21	228	182	228	180	1483	740	78	649	105	
Zagaia	-21.316666	-47.616666	21	228	182	228	180	1483	740	78	649	105	
Canastra	-20.35	-46.633	19.5	211	172	213	167	1483	779	52	617	75	
Canastra	-20.35	-46.633	19.5	211	172	213	167	1483	779	52	617	75	
Canastra	-20.35	-46.633	19.5	211	172	213	167	1483	779	52	617	75	
Canastra	-20.35	-46.633	19.5	211	172	213	167	1483	779	52	617	75	
Canastra	-20.35	-46.633	19.5	211	172	213	167	1483	779	52	617	75	
Canastra	-20.35	-46.633	19.5	211	172	213	167	1483	779	52	617	75	
Vassununga	-20.35	-46.3333	20.6	224	179	226	174	1411	749	48	595	71	
Vassununga	-20.35	-46.3333	20.6	224	179	226	174	1411	749	48	595	71	
Vassununga	-20.35	-46.3333	20.6	224	179	226	174	1411	749	48	595	71	

Vassununga	-20.35	-46.3333	20.6	224	179	226	174	1411	749	48	595	71	
Vassununga	-20.35	-46.3333	20.6	224	179	226	174	1411	749	48	595	71	
Vassununga	-20.35	-46.3333	20.6	224	179	226	174	1411	749	48	595	71	
Furnas	-20.2333333	-47.45	20.5	217	185	217	181	1543	781	43	781	73	
Furnas	-20.2333333	-47.45	20.5	217	185	217	181	1543	781	43	781	73	
Furnas	-20.2333333	-47.45	20.5	217	185	217	181	1543	781	43	781	73	
Furnas	-20.2333333	-47.45	20.5	217	185	217	181	1543	781	43	781	73	
Furnas	-20.2333333	-47.45	20.5	217	185	217	181	1543	781	43	781	73	
CA5	-7.45	-36.26667	23.1	236	230	244	211	369	199	13	131	91	
CA6	-7.45	-36.26667	23.1	236	230	244	211	369	199	13	131	91	
CA9	-7.45	-36.26667	23.1	236	230	244	211	369	199	13	131	91	
CB1	-7.4	-36.316667	23.1	236	231	244	211	373	206	13	138	86	
CB3	-7.4	-36.316667	23.1	236	231	244	211	373	206	13	138	86	
CB4	-7.4	-36.316667	23.1	236	231	244	211	373	206	13	138	86	
CB10	-7.4	-36.316667	23.1	236	231	244	211	373	206	13	138	86	
CB7	-7.4	-36.316667	23.1	236	231	244	211	373	206	13	138	86	
CB6	-7.4	-36.316667	23.1	236	231	244	211	373	206	13	138	86	
CC2	-7.116667	-36.133333	22.1	226	222	234	202	427	216	18	140	123	
CC5	-7.116667	-36.133333	22.1	226	222	234	202	427	216	18	140	123	
PA10	-29.883333	-55.583333	18.6	184	136	240	136	1635	463	361	400	361	
PA6	-29.883333	-55.583333	18.6	184	136	240	136	1635	463	361	400	361	
PA3	-29.883333	-55.583333	18.6	184	136	240	136	1635	463	361	400	361	BRAZIL/ (Machado de Lima et al., 2021)
PA2	-29.883333	-55.583333	18.6	184	136	240	136	1635	463	361	400	361	
PA1	-29.883333	-55.583333	18.6	184	136	240	136	1635	463	361	400	361	
PB5	-29.716667	-55.533333	18.7	186	137	241	137	1669	475	365	404	365	
PB10	-29.716667	-55.533333	18.7	186	137	241	137	1669	475	365	404	365	
PB8	-29.716667	-55.533333	18.7	186	137	241	137	1669	475	365	404	365	
PB7	-29.716667	-55.533333	18.7	186	137	241	137	1669	475	365	404	365	
PB6	-29.716667	-55.533333	18.7	186	137	241	137	1669	475	365	404	365	
PC10	-29.46667	-55.466667	18.9	187	140	242	140	1702	485	372	407	372	
PC9	-29.46667	-55.466667	18.9	187	140	242	140	1702	485	372	407	372	
PC8	-29.46667	-55.466667	18.9	187	140	242	140	1702	485	372	407	372	
PC6	-29.46667	-55.466667	18.9	187	140	242	140	1702	485	372	407	372	
PC4	-29.46667	-55.466667	18.9	187	140	242	140	1702	485	372	407	372	
PC3	-29.46667	-55.466667	18.9	187	140	242	140	1702	485	372	407	372	

PC2	-29.46667	-55.466667	18.9	187	140	242	140	1702	485	372	407	372	

Supplementary Table S2. Pairwise permanova results for cyanobacterial biocrust communities. Comparisons were calculated on the rarefied Bray-Curtis matrix. Purple font indicates those locations (pairs) where cyanobacterial communities were statistically different.

**Group 1**	**Group 2**	**Pseudo-F**	***P***
Mayapo 1	Mayapo 2	12.27	0.028
Mayapo 1	Murillo	7.31	0.109
Mayapo 1	Riohacha 1	31.03	0.092
Mayapo 1	Riohacha 2	47.66	0.098
Mayapo 1	Hoyos 1	16.57	0.093
Mayapo 1	Hoyos 2	22.19	0.103
Mayapo 1	Cusco 1	4.03	0.101
Mayapo 1	Cusco 2	17.07	0.113
Mayapo 2	Murillo	5.82	0.037
Mayapo 2	Riohacha 1	12.94	0.026
Mayapo 2	Riohacha 2	14.86	0.06
Mayapo 2	Hoyos 1	9.76	0.028
Mayapo 2	Hoyos 2	8.06	0.034
Mayapo 2	Cusco 1	3.42	0.025
Mayapo 2	Cusco 2	9.89	0.031
Murillo	Riohacha 1	7.03	0.103
Murillo	Riohacha 2	7.94	0.107
Murillo	Hoyos 1	5.44	0.096
Murillo	Hoyos 2	6.28	0.124
Murillo	Cusco 1	2.06	0.103
Murillo	Cusco 2	5.52	0.101
Riohacha 1	Riohacha 2	1.21	0.597
Riohacha 1	Hoyos 1	15.48	0.096
Riohacha 1	Hoyos 2	20.93	0.112
Riohacha 1	Cusco 1	3.92	0.1
Riohacha 1	Cusco 2	15.92	0.093
Riohacha 2	Hoyos 1	19.26	0.092
Riohacha 2	Hoyos 2	27.87	0.095
Riohacha 2	Cusco 1	4.29	0.09
Riohacha 2	Cusco 2	19.91	0.094
Hoyos 1	Hoyos 2	10.5	0.089

Hoyos 1	Cusco 1	1.87	0.097
Hoyos 1	Cusco 2	4.20	0.083
Hoyos 2	Cusco 1	1.91	0.118
Hoyos 2	Cusco 2	11.47	0.107
Cusco 1	Cusco 2	1.69	0.301

Supplementary Table S3. Bacterial/Archaeal community composition of Colombian biocrusts communities. Table includes mean and variance among replicates.

BACTERIAL/ARCHAEAL COMMUNITIES																				
	GUAJIRA DESERT																			
	Riohacha										Mayapo									
	R1					R2					Mayapo1					Mayapo 2				
Phyla	1	2	3	Average	SD	1	2	3	Average	SD	1	2	3	Average	SD	1	2	3	Average	SD
Cyanobacteria	501	549	390	480.00	81.55	304	369	1575	749.33	715.79	1696	2917	3680	2764.33	1000.77	5016	2916	5159	1255.75	1255.75
Chloroflexi	402	301	219	307.33	91.66	209	172	275	218.67	52.18	225	370	379	324.67	86.43	141	427	376	152.55	152.55
Firmicutes	317	248	143	236.00	87.62	109	146	345	200.00	126.93	152	295	746	397.67	310.02	328	28	53	166.46	166.46
Acidobacteria	135	93	64	97.33	35.70	40	95	56	63.67	28.29	91	65	364	173.33	165.63	169	711	438	271.00	271.00
Actinobacteria	32	46	35	37.67	7.37	7	8	400	138.33	226.61	277	116	175	189.33	81.45	241	781	235	313.52	313.52
Bacteroidetes	486	353	515	451.33	86.38	392	545	405	447.33	84.83	293	514	291	366.00	128.18	194	529	276	174.62	174.62
Proteobacteria	2233	2461	2679	2457.67	223.02	1732	2407	1027	1722.00	690.05	766	685	761	737.33	45.39	399	550	428	80.13	80.13
Verrucomicrobia	77	72	58	69.00	9.85	56	104	0	53.33	52.05	0	0	23	7.67	13.28	14	95	21	44.88	44.88
Planctomycetes	218	254	165	212.33	44.77	112	281	0	131.00	141.46	4	0	0	1.33	2.31	4	22	3	10.69	10.69
Gemmatimonadetes	12	28	27	22.33	8.96	15	25	107	49.00	50.48	66	32	68	55.33	20.23	67	54	12	28.75	28.75
Armatimonadetes	0	7	2	3.00	3.61	0	7	6	4.33	3.79	0	0	0	0.00	0.00	20	3	7	8.89	8.89
Elusimicrobia	0	0	0	0.00	0.00	0	0	0	0.00	0.00	0	0	0	0.00	0.00	0	0	0	0.00	0.00
Deinococcales	8	11	0	6.33	5.69	4	10	3	5.67	3.79	0	0	4	1.33	2.31	72	68	51	11.15	11.15
Chlamydiae	0	0	3	1.00	1.73	0	0	0	0.00	0.00	0	4	0	1.33	2.31	0	0	0	0.00	0.00
Chlorobi	0	0	0	0.00	0.00	0	6	0	2.00	3.46	0	0	0	0.00	0.00	0	2	0	1.15	1.15
Spirochaetes	9	5	5	6.33	2.31	10	18	0	9.33	9.02	0	0	0	0.00	0.00	0	0	0	0.00	0.00
Archaea	554	501	107	387.33	244.22	143	127	0	90.00	78.35	0	0	18	6.00	10.39	0	87	0	50.23	50.23
Other bacteria	31	53	22	35.33	15.95	17	37	150	68.00	71.71	44	188	458	230.00	210.17	359	395	268	65.45	65.45

	TATACOA DESERT																			
	Los Hoyos										Cusco									
	Los Hoyos 1					Los Hoyos 2					Cusco 1					Cusco 2				
Phyla	1	2	3	Average	SD	1	2	3	Average	SD	1	2	3	Average	SD	1	2	3	Average	SD
Cyanobacteria	3147	3236	588	2323.67	1503.79	2167	2171	2521	2286.33	203.24	2902	2874	1053	2276.33	1059.53	2555	2530	3465	2850.00	532.75
Chloroflexi	478	618	443	513.00	92.60	279	417	439	378.33	86.73	620	159	215	331.33	251.56	387	423	284	364.67	72.14
Firmicutes	5	0	279	94.67	159.66	114	283	208	201.67	84.68	32	105	0	45.67	53.82	0	0	0	0.00	0.00
Acidobacteria	368	521	85	324.67	221.21	156	222	151	176.33	39.63	835	110	133	359.33	412.10	598	568	291	485.67	169.25
Actinobacteria	180	286	31	165.67	128.10	316	352	283	317.00	34.51	287	388	530	401.67	122.08	363	441	477	427.00	58.28
Bacteroidetes	171	206	464	280.33	160.02	272	391	388	350.33	67.86	412	302	326	346.67	57.84	262	337	246	281.67	48.58
Proteobacteria	537	782	2193	1170.67	893.80	619	890	693	734.00	140.07	1289	632	966	962.33	328.52	665	650	552	622.33	61.37
Verrucomicrobia	70	169	162	133.67	55.25	257	548	332	379.00	151.09	456	83	264	267.67	186.53	990	523	211	574.67	392.06
Planctomycetes	44	70	376	163.33	184.63	59	68	45	57.33	11.59	110	4	76	63.33	54.12	53	114	8	58.33	53.20
Gemmatimonadetes	34	39	38	37.00	2.65	44	23	73	46.67	25.11	46	46	69	53.67	13.28	21	61	50	44.00	20.66
Armatimonadetes	7	42	0	16.33	22.50	24	51	18	31.00	17.58	30	7	22	19.67	11.68	38	42	18	32.67	12.86
Elusimicrobia	6	0	0	2.00	3.46	0	0	0	0.00	0.00	0	2	0	0.67	1.15	15	3	0	6.00	7.94
Deinococcales	0	0	11	3.67	6.35	0	9	4	4.33	4.51	11	3	36	16.67	17.21	0	0	0	0.00	0.00
Chlamydiae	0	0	3	1.00	1.73	0	0	0	0.00	0.00	0	0	0	0.00	0.00	0	0	0	0.00	0.00
Chlorobi	0	30	0	10.00	17.32	0	0	0	0.00	0.00	4	0	3	2.33	2.08	7	0	0	2.33	4.04
Spirochaetes	0	0	13	4.33	7.51	0	0	0	0.00	0.00	0	0	0	0.00	0.00	0	0	0	0.00	0.00
Archaea	62	46	553	220.33	288.21	2	0	0	0.67	1.15	73	40	0	37.67	36.56	72	74	0	48.67	42.16
Other bacteria	230	184	57	157.00	89.60	88	76	92	85.33	8.33	102	48	110	86.67	33.72	311	207	75	197.67	118.28

	TOLIMA				
	Murillo				
Phyla	1	2	3	Average	SD
Cyanobacteria	552	485	4189	1742.00	2119.43
Chloroflexi	152	112	1114	459.33	567.31
Firmicutes	0	2	0	0.67	1.15
Acidobacteria	399	1911	641	983.67	812.16
Actinobacteria	120	78	380	192.67	163.59
Bacteroidetes	355	363	351	356.33	6.11
Proteobacteria	1720	1400	975	1365.00	373.73
Verrucomicrobia	476	401	208	361.67	138.26
Planctomycetes	335	167	50	184.00	143.26
Gemmatimonadetes	0	21	46	22.33	23.03
Armatimonadetes	68	139	25	77.33	57.57
Elusimicrobia	0	0	11	3.67	6.35
Deinococcales	0	0	0	0.00	0.00
Chlamydiae	9	10	20	13.00	6.08
Chlorobi	0	0	23	7.67	13.28
Spirochaetes	35	0	0	11.67	20.21
Archaea	0	0	54	18.00	31.18
Other bacteria	434	223	186	281.00	133.79

Supplementary Table S4. Cyanobacterial community composition of Colombian biocrusts communities. Table includes mean and variance among replicates.

	CYANOBACTERIAL COMMUNITIES																			
	GUAJIRA DESERT																			
	Riohacha										Mayapo									
	R1					R2					Mayapo1					Mayapo 2				
**Species**	**1**	**2**	**3**	**Average**	**SD**	**1**	**2**	**3**	**Average**	**SD**	**1**	**2**	**3**	**Average**	**SD**	**1**	**2**	**3**	**Average**	**SD**
Scytonema	2.40	0.00	0.00	0.80	1.38	0.00	0.00	0.00	0.00	0.00	0.00	0.00	0.00	0.00	0.00	0.22	0.20	0.00	0.12	0.12
Wollea	8.58	8.33	6.01	7.64	1.42	10.51	8.22	9.49	9.41	1.15	0.00	0.00	0.00	0.00	0.00	0.00	0.00	0.00	0.00	0.00
Anabaena	16.37	20.75	21.49	19.54	2.77	30.26	32.57	21.14	27.99	6.04	0.00	0.00	0.00	0.00	0.00	0.00	0.00	0.00	0.00	0.00
**Other Nostocales**	0.00	3.74	2.91	2.22	1.97	2.82	0.00	2.71	1.84	1.60	6.10	4.79	6.27	5.72	0.81	0.00	0.00	0.21	0.12	0.12
Potamolinea	0.00	0.00	0.00	0.00	0.00	0.00	0.00	0.00	0.00	0.00	61.59	54.55	50.77	55.64	5.49	0.46	0.00	4.22	2.31	2.31
Parifulum	0.00	0.00	0.00	0.00	0.00	0.00	0.00	0.00	0.00	0.00	1.78	1.65	0.00	1.14	0.99	41.36	40.21	28.81	6.94	6.94
Pycnacronima	0.00	0.00	0.00	0.00	0.00	0.00	0.00	0.00	0.00	0.00	0.00	0.00	0.00	0.00	0.00	1.03	0.22	4.73	2.41	2.41
Porphyrosiphon	0.00	0.00	0.00	0.00	0.00	0.00	0.00	0.00	0.00	0.00	0.00	0.00	0.00	0.00	0.00	0.00	0.00	0.00	0.00	0.00
Arizonema	0.00	0.00	0.00	0.00	0.00	0.00	0.00	0.00	0.00	0.00	0.00	0.00	0.00	0.00	0.00	0.00	0.00	0.00	0.00	0.00
C. chtonoplastes	0.00	0.00	0.00	0.00	0.00	0.00	0.00	0.00	0.00	0.00	0.00	0.77	0.00	0.26	0.44	0.00	0.00	0.00	0.00	0.00
Capilliphycus	47.11	48.64	42.08	45.94	3.43	39.49	47.04	53.39	46.64	6.96	0.00	0.00	0.00	0.00	0.00	0.00	0.00	0.00	0.00	0.00
Leptolyngbya	12.97	15.48	17.30	15.25	2.17	15.38	11.18	0.00	8.86	7.95	8.44	9.69	10.76	9.63	1.16	1.68	0.54	0.00	0.86	0.86
Planktothrix	0.00	0.00	0.00	0.00	0.00	0.00	0.00	0.00	0.00	0.00	7.37	7.39	14.26	9.67	3.97	0.00	0.00	0.00	0.00	0.00
M. vaginatus	0.00	0.00	0.00	0.00	0.00	0.00	0.00	0.00	0.00	0.00	0.00	0.00	0.00	0.00	0.00	0.00	0.00	0.00	0.00	0.00
Oscillatoria	0.00	0.00	0.00	0.00	0.00	0.00	0.00	0.00	0.00	0.00	0.00	0.00	0.00	0.00	0.00	0.00	0.00	0.00	0.00	0.00
Lyngbya	0.00	0.00	0.00	0.00	0.00	0.00	0.00	0.00	0.00	0.00	0.00	2.48	0.00	0.83	1.43	0.00	0.00	0.00	0.00	0.00
Schizothrix	0.00	0.00	0.00	0.00	0.00	0.00	0.00	0.00	0.00	0.00	0.00	0.00	0.00	0.00	0.00	42.34	54.53	10.97	22.47	22.47
**Chroococcales**	0.00	0.00	0.00	0.00	0.00	0.00	0.99	0.00	0.33	0.57	0.00	0.00	0.00	0.00	0.00	0.00	0.00	0.00	0.00	0.00
**Synechococcales**	0.00	0.00	0.00	0.00	0.00	0.00	0.00	0.00	0.00	0.00	0.00	0.89	0.34	0.41	0.45	0.00	0.00	0.00	0.00	0.00
**Unassigned**	12.57	3.06	10.20	8.61	4.95	1.54	0.00	13.28	4.94	7.26	14.73	17.79	17.59	16.70	1.71	12.91	4.31	51.06	24.89	24.89

	TATACOA DESERT																			
	Los Hoyos										Cusco									
	Los Hoyos 1					Los Hoyos 2					Cusco 1					Cusco 2				
**Species**	**1**	**2**	**3**	**Average**	**SD**	**1**	**2**	**3**	**Average**	**SD**	**1**	**2**	**3**	**Average**	**SD**	**1**	**2**	**3**	**Average**	**SD**
Scytonema	52.45	49.60	37.86	46.64	7.74	9.09	6.13	5.67	6.96	1.86	68.81	2.40	45.30	38.84	33.67	38.51	49.57	83.15	57.07	23.24
Wollea	0.00	0.00	0.00	0.00	0.00	0.00	0.00	0.00	0.00	0.00	0.00	0.00	0.00	0.00	0.00	0.00	0.00	0.00	0.00	0.00
Anabaena	0.00	0.00	0.00	0.00	0.00	0.00	0.00	0.00	0.00	0.00	0.00	0.00	0.00	0.00	0.00	0.00	0.00	0.00	0.00	0.00
**Other Nostocales**	0.14	0.00	0.00	0.05	0.08	2.91	7.55	1.71	4.06	3.09	5.03	2.71	0.00	2.58	2.52	0.00	0.67	3.67	1.45	1.95
Potamolinea	1.86	0.00	1.21	1.02	0.94	0.00	0.88	0.91	0.60	0.52	0.00	0.42	0.00	0.14	0.24	0.00	0.79	0.00	0.26	0.46
Parifulum	14.18	16.97	34.12	21.75	10.80	8.68	11.75	11.07	10.50	1.61	1.72	28.36	0.00	10.03	15.90	5.60	6.01	1.65	4.42	2.41
Pycnacronima	0.00	0.00	1.48	0.49	0.86	1.75	1.52	2.74	2.00	0.65	0.00	8.73	0.00	2.91	5.04	0.00	0.00	0.00	0.00	0.00
Porphyrosiphon	0.00	1.30	2.07	1.12	1.05	0.00	0.00	0.00	0.00	0.00	0.00	0.00	0.00	0.00	0.00	0.00	0.00	0.92	0.31	0.53
Arizonema	0.00	0.00	0.00	0.00	0.00	1.02	0.00	4.24	1.75	2.22	0.00	0.00	0.00	0.00	0.00	0.00	0.00	0.00	0.00	0.00
C. chtonoplastes	0.00	0.00	0.00	0.00	0.00	0.00	0.00	0.00	0.00	0.00	0.00	0.00	0.00	0.00	0.00	0.00	0.00	0.00	0.00	0.00
Capilliphycus	0.00	0.00	0.00	0.00	0.00	0.00	0.00	0.00	0.00	0.00	0.00	0.00	0.00	0.00	0.00	0.00	0.00	0.00	0.00	0.00
Leptolyngbya	0.00	0.54	0.22	0.25	0.27	4.34	2.58	2.70	3.20	0.98	0.45	0.00	9.12	3.19	5.14	0.00	0.00	0.09	0.03	0.05
Planktothrix	0.00	0.00	0.00	0.00	0.00	0.00	0.00	0.00	0.00	0.00	0.00	0.00	0.00	0.00	0.00	0.00	0.00	0.00	0.00	0.00
M. vaginatus	0.24	3.59	0.00	1.28	2.01	0.00	1.52	0.24	0.59	0.82	0.00	19.55	1.80	7.12	10.81	0.20	0.00	2.02	0.74	1.11
Oscillatoria	0.00	0.00	0.00	0.00	0.00	0.00	0.00	0.00	0.00	0.00	0.00	0.00	0.00	0.00	0.00	0.20	0.00	0.00	0.07	0.11
Lyngbya	0.00	0.00	0.00	0.00	0.00	0.00	0.00	0.32	0.11	0.18	0.00	0.00	0.00	0.00	0.00	0.00	0.00	0.00	0.00	0.00
Schizothrix	13.99	1.24	3.15	6.13	6.88	0.00	0.00	0.32	0.11	0.18	0.00	0.00	0.00	0.00	0.00	0.00	1.26	0.00	0.42	0.73
**Chroococcales**	0.00	0.00	0.00	0.00	0.00	0.00	0.00	0.00	0.00	0.00	0.00	0.42	2.18	0.87	1.16	0.00	0.00	0.00	0.00	0.00
**Synechococcales**	0.00	0.00	0.00	0.00	0.00	0.00	0.00	0.00	0.00	0.00	0.00	0.00	0.47	0.16	0.27	0.12	0.24	0.00	0.12	0.12
**Unassigned**	17.14	26.76	19.90	21.27	4.95	72.22	68.08	70.09	70.13	2.07	23.98	37.40	41.12	34.17	9.01	55.38	41.46	8.51	35.12	24.07

	TOLIMA				
	Murillo				
Species	1	2	3	Average	SD
Scytonema	29.22	68.89	25.26	41.12	24.13
Wollea	0.00	0.00	0.00	0.00	0.00
Anabaena	0.00	0.00	0.00	0.00	0.00
Other Nostocales	14.76	10.37	20.76	15.30	5.22
Potamolinea	0.00	0.00	0.00	0.00	0.00
Parifulum	0.00	0.00	0.00	0.00	0.00
Pycnacronima	0.00	0.00	0.00	0.00	0.00
Porphyrosiphon	0.00	0.00	0.00	0.00	0.00
Arizonema	0.00	0.00	0.00	0.00	0.00
C. chtonoplastes	0.00	0.00	0.00	0.00	0.00
Capilliphycus	0.00	0.00	0.00	0.00	0.00
Leptolyngbya	31.93	0.00	39.10	23.68	20.82
Planktothrix	0.00	0.00	0.00	0.00	0.00
M. vaginatus	0.00	0.00	0.00	0.00	0.00
Oscillatoria	0.00	0.00	0.00	0.00	0.00
Lyngbya	0.00	0.00	0.00	0.00	0.00
Schizothrix	0.00	0.00	0.00	0.00	0.00
Chroococcales	0.00	0.00	0.00	0.00	0.00
Synechococcales	0.00	0.00	0.00	0.00	0.00
Unassigned	24.10	20.74	14.88	19.91	4.67

References

Becerra-Absalón, I., Muñoz-Martín, M. Á., Montejano, G., and Mateo, P. (2019). Differences in the Cyanobacterial Community Composition of Biocrusts From the Drylands of Central Mexico. Are There Endemic Species? *Front. Microbiol.* 10, 1–21. doi: 10.3389/fmicb.2019.00937.

Couradeau, E., Karaoz, U., Lim, H. C., Nunes da Rocha, U., Northen, T., Brodie, E., et al. (2016). Bacteria increase arid-land soil surface temperature through the production of sunscreens. *Nat. Commun.* 7, 1–7. doi: 10.1038/ncomms10373.

Fernandes, V. M. C., Machado de Lima, N. M., Roush, D., Rudgers, J., Collins, S. L., and Garcia-Pichel, F. (2018). Exposure to predicted precipitation patterns decreases population size and alters community structure of cyanobacteria in biological soil crusts from the Chihuahuan Desert. *Environ. Microbiol.* 20, 259–269. doi: 10.1111/1462-2920.13983.

Giraldo-Silva, A., Nelson, C., Barger, N., and Garcia-Pichel, F. (2019). Nursing biocrusts: isolation, cultivation and fitness test of indigenous cyanobacteria. *Restor. Ecol.* 27, 793–803. doi: 10.1111/rec.12920.

Machado de Lima, N. M., Fernandes, V. M. C., Roush, D., Velasco Ayuso, S., Rigonato, J., Garcia-Pichel, F., et al. (2019). The Compositionally Distinct Cyanobacterial Biocrusts From Brazilian Savanna and Their Environmental Drivers of Community Diversity. *Front. Microbiol.* 10, 1–10. doi: 10.3389/fmicb.2019.02798.

Machado de Lima, N. M., Muñoz-Rojas, M., Vázquez-Campos, X., and Branco, L. H. Z. (2021). Biocrust cyanobacterial composition, diversity, and environmental drivers in two contrasting climatic regions in Brazil. *Geoderma* 386. doi: 10.1016/j.geoderma.2020.114914.

Nelson, C., Giraldo-silva, A., Thomas Warsop, F., and Garcia-Pichel, F. (2022). Spatial self-segregation of pioneer cyanobacterial species drives microbiome organization in biocrusts. 1–9. doi: 10.1038/s43705-022-00199-0.
